# Supplementary material for: Stomach Cancer and Exposure to Talc Powder without Asbestos via Chinese Herbal Medicine: A Population-Based Cohort Study
Source: Int J Environ Res Public Health. 2019 Feb 28;16(5):717. doi: 10.3390/ijerph16050717 (PMC6427112; doi:10.3390/ijerph16050717)
Supplement: Supplementary file 1 [file ijerph-16-00717-s001.pdf]

Supplementary materials

Table of contents

Appendix 1. SAS main script 2
Appendix 2. SAS modules scripts 8
ID list.sas 8
Exclusion_cancer_mark.sas 9
Exclusion_hpylori.sas 11
Drug_containing_prescription.sas 12
Demographic_analysis.sas 13
CCI calculation.sas 15
Chinese_herb_process.sas 17
Summing_exposure.sas 18
Level_of_dose.sas 19
Time_dependent_exposure.sas 20
Before_regression_process.sas 22
Num_of_event_table.sas 23
Cox_regression_of_talc.sas 24
Appendix 3. Drug codes of Chinese herbal medicine containing talc 27
Appendix 4. Level of urbanization according to the region code in Taiwan 29

Appendix 1. SAS main script

Filename: Main script.sas

Description: The SAS main script for the process of statistical analysis using SAS 9.4. The main script calls other SAS modules listed in Appendix 2.

```
    # Talc Exposure and Risk of Stomach Cancer: Systemic review and Meta-Analysis of Occupational Cohort Studies
    # set-up:
/*
Stomach Cancer and Exposure to Non-asbestiform Talc Powder via Chinese Herbal Medicine: A Population-based
Cohort Study
Time: January 1st 1997 ~ December 31st 2013
Patients: LHID 2005
Intervention: talc inake from Chinese herbal medicine
Comparison: patients in LHID 2005 without talc intake
Outcome: Diagnosis of stomach cancer
*/

/*OPTIONS settings*/
options nolabel nonumber nodate mprint;     /*
                                                NOLABEL ---  not using labels with variables
                                                NONUMBER --- not printing the page number
                                                NODATE --- the date and the time are not printed
                                                MPRINT --- displays the SAS statements that are generated by
macro execution */

/*Data library settings*/
libname talc "\talc\";     /*directory for data output */
libname M "\main datasets\";            /*directory for data input from LHID 2005 */
libname drug "\supplement datasets\";   /*directory for data regarding drug codes and urbanization levels*/

/*Setting directory of SAS modules*/
%let basepath = \SAS modules\;


/* Constructing the "ID List" of target population*/
%include "&basepath.id list first id_20180818.sas";

/* Data exclusion:
The exclusion criteria were:
            - patients younger than 20,
            - patients with diagnosis of cancer in 1997,
            - patients with diagnosis of H.pylori / Peptic ulcer disease / Duodenal ulcer / Gastric ulcer / Gastritis /
Duodenitis,
Parameters:
            lib_from: library containing HV files (Registry for catastrophic illness patients)
            cancer_icd: icd-9-cm code of the cancer of interest (stomach cancer in our case)
            ID_list: target file containing the "ID list" to be marked for cancer diagnosis
```

```
*/
%include "&basepath.exclusion_cancer_mark.sas";
%exclusion_cancer_mark(m, '151', talc.ID_list); /*the ICD-9-CM code of stomach cancer is "151" */
%include "&basepath.exclusion_hpylori.sas";
%exclusion_hpylori(talc.ID_list); /*Excluding patients with H.pylori / Peptic ulcer disease / Duodenal ulcer / Gastric
ulcer / Gastritis / Duodenitis */


/*Import the drug number of talc-containing medication*/
DATA drug.talc_chinese; SET drug.talc_chinese; RUN;

/*Finding the medical talc prescription data*/
%include "&basepath.drug_containing_prescription.sas";
/* using the drug code to identify all ambulatory care orders containing the target medication,
and store the results in the dataset "ooCE"*/
        /* Parameters
        (OO = the NHIRD dataset containing the details of ambulatory care orders)
        lib_from: the library where OO files exist.
        file_drug: the file with required drug number
        lib_out: the library to output the target file ooCE
        year_from: starting year of OO files
        year_to: ending year of OO files
        */
%drug_containing_prescription_1(m, drug.talc_chinese, talc, 1997, 2013);
/*Using the "ooCE" dataset (ambulatory care orders containing target medication) to identify corresponding CD files,
in order to find out the ID and the diagnoses*/
        /*Parameters
        (CD = the NHIRD dataset containing the ambulatory care expenditures by visits)
        lib_from: the library where CD files exist.
        lib_out: the library to output the target file cdooCE
        year_from: starting year of CD files
        year_to: ending year of CD files
        */
%drug_containing_prescription_2(m, talc, 1997, 2013);

/*Pooled together all medical talc prescription data, and sort by the ID*/
Data talc.exposure_data;          set talc.cdooCE1997-talc.cdooCE2013;
proc sort;by ID;    run;

/*View the main diagnoses corresponding to the medical talc prescription data*/
PROC SORT Data = talc.exposure_data;          /*sort the medical talc prescription data by drugs and then by
ID*/
BY talc_frc id; RUN;
DATA talc.icd9main_exposure; SET talc.exposure_data; /*icd9main_exposure: containing the main diagnosis
(dx_exposure) of each prescription*/
dx_exposure = substr(icd9_1, 1, 3); RUN;
PROC FREQ; /*icd9_by_drug: output table of the main diagnosis of each prescription by drugs*/
BY talc_frc;
TABLES dx_exposure / norow nopercent out = talc.icd9_by_drug;
RUN;
```

```
PROC SORT Data = talc.icd9_by_drug;          /*to identify the most frequent diagnosis of talc-containing
prescription*/
BY talc_frc descending count; RUN;

/*Pre-process of analyzing the demographics (Table 1)*/
/*adding variable "ever_talc": whether the patient ever exposed to talc*/
PROC SQL; CREATE TABLE talc.ID_list as
SELECT *, case
        when id in (select id from talc.exposure_data) then 1
        else 0
        end as ever_talc
FROM talc.ID_list; QUIT;


/*Table 1.demographics: talc-exposed vs. unexposed
        age (at the start date of the study)
        sex (gender of the beneficiary)
        income (monthly income in TWD)
        fu_time: follow-up time
        urban: level of urbanization (high, medium, low)
*/
        /* Paremeters:
                ID_list: target file containing the ID list
                depend: dependent variable for grouping
        */
%include "&basepath.demographic_analysis.sas";
%demographic_analysis(talc.ID_list, ever_talc);


/*Checking the cross table of "ever exposed to talc" and "diagnosis of stomach cancer"*/
proc freq data = talc.id_list;
tables ca * ever_talc  / norow nopercent chisq;  run;


/*statistics of the follow-up time; summing up the follow-up person-year*/
PROC MEANS data = talc.id_list N MIN MEAN STD MAX Q1 MEDIAN Q3 SUM;
var fu_time; run;


/*==============================================*/
/*Calculate the Charlson Comorbidity Index (CCI)*/
/* Calculate the CCI based on icd-9-cm codes (and three visits with the same diagnosis in one year)*/
           /*  dbname: name of the library containing records of OPD visits
               outfile: the target file for marking the diagnoses*/
%include "&basepath.CCI calculation.sas";
%dx2times(talc.ID_list);
Proc Freq data = talc.ID_list; tables Charlson * ever_talc; run; /*Check the relationship between Chalson score (CCI)
and exposure ot talc*/
Data talc.ID_list; Set talc.ID_list; /*Define Charlson > 2 as high CCI*/
        if not (Charlson > 0) then Charlson = 0; /* missing value --> 0*/
```

```
		if Charlson <= 2 then CCI = "low ";			else CCI = "high";
run;
/*Check the relationship between levels of Chalson score (high CCI and low CCI) and exposure ot talc*/
proc freq; tables CCI * ever_talc  / norow nopercent chisq;		run;


/* Assessing the amount of talc exposure and dealing with the missing value*/
	/*Parameter:
		drug_data: dataset with drug prescription data
	*/
%include "&basepath.chinese_herb_process.sas";
%chinese_herb_process(talc.exposure_data);


/*Before the "time-dependent process", sum up the exposure and compare between cancer vs. non-cancer*/
	/*Parameters
		ID_list: target file containing the ID list
		drug_data: dataset with drug prescription data
		output_file: the name of the resulting output file
	*/
%include "&basepath.summing_exposure.sas";
%summing_exposure(talc.ID_list, talc.exposure_data, ID_list);

/*Check median and quartile for talc exposure*/
PROC MEANS data = ID_LIST RANGE Q1 MEDIAN Q3 MEAN SUM;
var sum_dose; RUN; /*the results: Q1 = 6, Median = 10.5, Q3 = 21*/

/*(before the "time-dependent process") grouping of the talc exposure into 3 levels*/
	/* Parameters:
		target_file: the target dataset for Cox regression
		scalar: scalar variable to be categorized by the cut-off values
		cutoff01: cut-off point 01
		cutoff02: cut-off point 02
		level: categorical variable resulting from the cut-offs
		depend: depending variable
	*/
%include "&basepath.level_of_dose.sas";
%level_of_dose(ID_list, sum_dose, 6, 21, level_dose, ca); /*(levels of talc exposure) low: <6, median: 6~21, high:
>21*/

/*"time-dependent process": We treated the talc exposure as a time-dependent variable
in order to eliminate the immortal time bias, which is a form of selection bias arising
when the period between cohort entry and date of first exposure to a drug is either misclassified
or simply excluded because the event of interest has not occurred*/
	/*Parameter
		drug_data: dataset with drug prescription data
	*/
%include "&basepath.time_dependent_exposure.sas";
%time_dependent_exposure(talc.exposure_data); /*For patients ever received medical prescription of talc,
we considered the time interval between the beginning of the study (January 1st 1997) and the date of
```

```
first prescription of talc to be a non-exposure period, whereas the time interval from the date of first prescription
of talc to the endpoint of follow-up was recognized as an exposure period. */

/*Procedures before doing the regression*/
	/*Mapping the exposure data to the target cohort (the "id_list")*/
	/*For each time period: set up the event status and the time-to-event*/
	/*For people without talc exposure, set up a non-exposure period from Jan/01/1997 to follow-up end date
(fu_date)*/
	/*ever_talc (whether she had ever taken talc) needs to be rechecked*/
	/*fu_time (follow-up time) needs to be re-calculated*/
	/*if the time period is not the last for that person, set the follow-up endpoint (fu_date) to the end date of that
period (drug_end_date)*/
	/*defining "elder" as age >= 65*/
	/* Parameters:
	ID_list: target file containing the ID list
	drug_data: dataset with drug prescription data
	output_file: the name of the resulting output file for subsequent regression
	*/
%include "&basepath.before_regression_process.sas";
%before_regression_process(talc.ID_list, talc.exposure_data, talc.before_regression);



/*(after the "time-dependent process") grouping of the talc exposure into 3 levels*/
	/* Parameters:
		target_file: the target dataset for Cox regression
		scalar: scalar variable to be categorized by the cut-off values
		cutoff01: cut-off point 01
		cutoff02: cut-off point 02
		level: categorical variable resulting from the cut-offs
		depend: depending variable
	*/
%include "&basepath.level_of_dose.sas";
%level_of_dose(talc.before_regression, sum_dose, 6, 21, level_dose, ca);


/*Reporting numbers of outcome events or summary measures over time.*/
	/*Parameters
		ID_list: target file containing the ID list to calculate the of outcome events
		event: the name of variable of interest (the event)
		fu_time_year: the variable name containing the follow-up time (year)
	*/
%include "&basepath.num_of_event_table.sas";
%num_of_event_table(talc.before_regression);


/*2018.01 Cox regression of talc
dependent variable: diagnosis of stomach cancer (ca)
explanatory variables: ever exposure to talc (ever_talc), level of talc exposure (level_dose)
confounders: age, gender, CCI*/
```

```
	/* Parameter:
		ID_list: target file containing the time, the outcome, and the variables.
	*/
%include "&basepath.Cox_regression_of_talc.sas";
%Cox_regression_of_talc(talc.before_regression);



/*Sensitivity analysis: Cut-off points of talc exposure*/
	/*alternative way of grouping of the talc exposure into 3 levels (unexposed, ? median, > median)*/
	Data talc.before_regression_S1; Set talc.before_regression; Run;
	%include "&basepath.level_of_dose.sas";
	%level_of_dose(talc.before_regression_S1, sum_dose, 0, 10.5, level_dose, ca);
	/*Report numbers of outcome events or summary measures over time.	*/
	%include "&basepath.num_of_event_table.sas";
	%num_of_event_table(talc.before_regression_S1);
	/*Cox regression of talc*/
	%include "&basepath.Cox_regression_of_talc.sas";
	%Cox_regression_of_talc(talc.before_regression_S1);


/*Sensitivity analysis: Minimal induction period*/
	Data talc.before_regression_S2; Set talc.before_regression;
	if time < 5 then delete;			Run;  /*delete all follow-up time < 5 years, to make sure minimal
induction period to be at least 5 years*/
	%include "&basepath.num_of_event_table.sas";
	%num_of_event_table(talc.before_regression_S2);
	/*Cox regression of talc*/
	%include "&basepath.Cox_regression_of_talc.sas";
	%Cox_regression_of_talc(talc.before_regression_S2);

/*Sensitivity analysis: CCI*/
	/*Redefine CCI>0 as high comorbidities*/
	Data talc.before_regression_S3; Set talc.before_regression;
	if Charlson > 0 then CCI = "high";		else CCI = "low ";
	Run;
	%include "&basepath.num_of_event_table.sas";
	%num_of_event_table(talc.before_regression_S3);
	/*Cox regression of talc*/
	%include "&basepath.Cox_regression_of_talc.sas";
	%Cox_regression_of_talc(talc.before_regression_S3);
	Data talc.ID_list_S3; Set talc.ID_list;
	if Charlson > 0 then CCI = "high";		else CCI = "low ";
	run;
	/*Checking the relationship between level of CCI and ever exposure to talc*/
	proc freq; tables CCI * ever_talc  / norow nopercent chisq;		run;
```

Appendix 2. SAS modules scripts

Filename:

ID list.sas

Exclusion_cancer_mark.sas

Drug_containing_prescription.sas

Demographic_analysis.sas

CCI calculation.sas

Chinese_herb_process.sas

Summing_exposure.sas

Level_of_dose.sas

Time_dependent_exposure.sas

Before_regression_process.sas

Num_of_event_table.sas

Cox_regression_of_talc.sas

Description: The SAS modules for certain functions in the analysis.

ID list.sas

```
/*
Constructing the "ID List" of target population
*/
data talc.ID_List;
set m.id2000 m.id2002-m.id2013; /*aggregate data from the Registry for beneficiaries (ID) */
keep ID INS_AMT ID_BIRTHDAY ID_SEX AREA_NO_I REG_ZIP_CODE ID_OUT_DATE;
	/* variables:
	ID: Beneficiary identification
	ID_BIRTHDAY: date of birth
	ID_SEX: gender
```

```
            AREA_NO_I: place of residence (old post code)
            REG_ZIP_CODE: place of residence (new post code)
            ID_OUT_DATE: date of cancellation of insurance
            */
run;
proc sort data = talc.ID_List;          by id; /*sort by id*/
run;
data ID_LastID;   set talc.ID_List;     by id; /*get the latest ID_OUT_DATE by extracting from the last entry by ID*/
if last.id then output;
run;
data talc.ID_List;  set talc.ID_List;   by id; /*remove duplicates by extracting from the first entry by ID*/
if first.id then output;
run;
PROC SQL; CREATE TABLE talc.ID_List(drop = x) AS /*update the "ID_list" with the latest ID_OUT_DATE*/
SELECT a.*, b.ID_OUT_DATE
FROM talc.ID_List(rename=(ID_OUT_DATE=x)) as a, ID_LASTID as b
WHERE a.ID = b.ID; QUIT;
```

Exclusion_cancer_mark.sas

```
/* Data exclusion:
The exclusion criteria were:
            - patients younger than 20,
            - patients with diagnosis of cancer in 1997,
Parameters:
            lib_from: library containing HV files (Registry for catastrophic illness patients)
            cancer_icd: icd-9-cm code of the cancer of interest (stomach cancer in our case)
            ID_list: target file containing the "ID list" to be marked for cancer diagnosis
*/

%macro exclusion_cancer_mark(lib_from, cancer_icd, ID_list);
/*excluding patients younger than 20*/
data &ID_list.; set &ID_list.;
if intck('year', id_birthday, mdy(1,1,1997)) < 20 then delete; /*calculating age (year) from Jan.01.1997; exclude if age
< 20*/
RUN;


/*Identify patients with diagnosis of cancer*/
data cancer_id;
set &lib_from..hv1997-&lib_from..hv2013; /*HV: Registry for catastrophic illness patients*/
keep ID icd9cm_code HV_TYPE APPL_DATE;
            /* Variables:
            ID: Holder identification
```

```
        icd9cm_code: diagnoses of catastrophic illness
        HV_TYPE: type of catastrophic illness (01 = cancer)
        APPL_DATE: date of application
        */
if HV_TYPE not in ('01') then delete; /*Identify patients with cancer*/
proc sort;by id;    run;

/*Identify patients with diagnosis of cancer of interest*/
DATA case_id;
SET cancer_id;
if substr( icd9cm_code,1,3) in (&cancer_icd.) then output case_id; /*keep the ID if icd-9-cm matches the cancer of
interest*/
proc sort;by id;    run;
DATA case_id;       SET case_id;    by id;    if first.id then output;      run; /*remove duplicates*/

/*Identify patients with diagnosis of cancer of interest AT THE BEGINNING of the study*/
DATA cancer_id_1997;
set &lib_from..hv1997;
keep ID icd9cm_code HV_TYPE APPL_DATE;
if HV_TYPE not in ('01') then delete;
proc sort;by id;    run;
data cancer_id_1997;       set cancer_id_1997;      by id;    if first.id then output;      run;

/*Excluding patients with cancer in 1997; stored in a temporary dataset*/
PROC SQL;  create table ID_LIST as
select * from &ID_LIST.
where id not in (select id from cancer_id_1997);
QUIT;

 /*Marking patients in id_list with diagnosis of cancer of interest (ca = 1),
and marking the date of that cancer (ca_date)*/
PROC SQL;           create table ID_LIST as
select a.*, 1 as ca, b.appl_date as ca_date
from ID_list as a LEFT JOIN case_id as b
on a.id = b.id;           QUIT;
proc sort data = ID_LIST;         by id;
run;
DATA &id_list.; SET id_list; /*if the date of the cancer exists, then he/she has cancer (ca = 1)*/
ca = ca_date;      if ca = '.' then ca = 0;       else ca = 1;
RUN;

%mend exclusion_cancer_mark;
```

Exclusion_hpylori.sas

```
/*Excluding patients with H.pylori / Peptic ulcer disease / Duodenal ulcer / Gastric ulcer / Gastritis / Duodenitis*/
	/*Prerequisite:
		D102 or the CD files (datasets of ambulatory care expenditures by visit)
	*/
	  /* input parameter:
				outfile: the target file containing the ID list
			ouput: modified &outfile by excluding targeted diagnoses
		*/

%MACRO dx2times(outfile);
	/*For m.cd1997 (ambulatory care expenditures by visit):
	determine the comorbidities based on the ICD codes
	output: work.dx1time
	*/
	DATA dx1time;
		set
		m.cd1997
		;
		array dxcode{3} icd9_1 icd9_2 icd9_3;
		do i= 1 to dim(dxcode);
			if dxcode(i) in :('531') then GU= 1;
			if dxcode(i) in :('532') then DU= 1;
			if dxcode(i) in :('533') then PUD= 1;
			if dxcode(i) in :('535') then Gastritis= 1;
			if dxcode(i) in :('04186') then Hpylori= 1;
		end;
		drop i; run;

	/*calculate the frequency of diagnoses: looking for >= 2 OPD visits*/
	proc means data=dx1time nway noprint;
	class id;
	var GU--Hpylori;
	output out=dx2times  sum = ;
	run;


	*** Select target patients ;
	DATA TARGET; SET dx2times;
	array comorb{*} GU--Hpylori;
	do i = 1 to dim(comorb);
		if comorb(i) >= 2 then comorb(i) = 1;  /*diagnosis criteria:  >= 2 OPD visits*/
		else comorb(i) = 0;
	end;
```

```
    drop i;
    if (GU = 1) OR (DU = 1) OR (PUD = 1) OR (Gastritis = 1) OR (Hpylori = 1) then output;
    RUN;

    /*Back to the outfile (the ID list)*/
    PROC SQL; CREATE TABLE &outfile. AS
        select *
        from &outfile.
        where id NOT in (select id from TARGET);
    QUIT;

%mend dx2times;
```

Drug_containing_prescription.sas

```
/* using the drug code to identify all ambulatory care orders containing the target medication,
and store the results in the dataset "ooCE"*/
    /* Parameters
    (OO = the NHIRD dataset containing the details of ambulatory care orders)
    lib_from: the library where OO files exist.
    file_drug: the file with required drug number
    lib_out: the library to output the target file ooCE
    year_from: starting year of OO files
    year_to: ending year of OO files
    */
%macro drug_containing_prescription_1(lib_from, file_drug, lib_out, year_from, year_to);
%do i=&year_from. %to &year_to.;
PROC SQL;
CREATE TABLE &lib_out..ooCE&i. AS                    /*stored in ooCE&i. for the results in each year
(denoted by i)  */
SELECT a.FEE_YM, a.HOSP_ID, a.APPL_TYPE, a.APPL_DATE, a.CASE_TYPE, a.SEQ_NO, a.total_qty,
a.drug_no, a.drug_use, a.drug_fre, b.talc_frc
FROM &lib_from..oo&i. as a, &file_drug. as b                          /*reading the OO file of each
year (denoted by i) */
WHERE a.drug_no = b.drug_no;
QUIT;
%end;
%mend drug_containing_prescription_1;

/*Using the "ooCE" dataset (ambulatory care orders containing target medication) to identify corresponding CD files,
in order to find out the ID and the diagnoses*/
    /*Parameters
    (CD = the NHIRD dataset containing the ambulatory care expenditures by visits)
    lib_from: the library where CD files exist.
```

```
	lib_out: the library to output the target file cdooCE
	year_from: starting year of CD files
	year_to: ending year of CD files
	*/
%macro drug_containing_prescription_2 (lib_from, lib_out, year_from, year_to);
%do i=&year_from. %to &year_to.;
PROC SQL;
CREATE TABLE &lib_out..cdooCE&i. AS
SELECT a.ID, a.icd9_1, a.icd9_2, a.icd9_3, a.drug_day, a.id_sex, a.func_date, b.*
FROM &lib_from..cd&i.  as a, &lib_out..ooCE&i. as b
WHERE a.FEE_YM=b.FEE_YM and a.hosp_id=b.hosp_id and a.APPL_TYPE=b.APPL_TYPE and
    a.APPL_DATE=b.APPL_DATE and a.CASE_TYPE=b.CASE_TYPE and a.SEQ_NO=b.SEQ_NO;
QUIT;
%end;
%mend drug_containing_prescription_2;
```

Demographic_analysis.sas

```
/*Table 1.demographics:  talc-exposed vs. unexposed
	age (at the start date of the study)
	sex (gender of the beneficiary)
	income (monthly income in TWD)
	fu_time: follow-up time
	urban: level of urbanization (high, medium, low) from the dataset "urban_3_level"
*/
	/* Paremeters:
		ID_list: target file containing the ID list
		depend: dependent variable for grouping
	*/
%macro demographic_analysis(ID_list, depend);
data &ID_list.;			set &ID_list.;
income = ins_amt; if income = 0 then income = '.'; /*ins_amt: monthly income (TWD); if income = 0, then the beneficiary is in the dependent population*/
age = intck('year', id_birthday, mdy(1,1,1997)); /*calculate age at the start date of the study*/
format fu_date date9.; /*fu_date: follow-up endpoint*/
if ca_date NE '.' then fu_date = ca_date; /*define follow-up endpoint (fu_date) as cancer date or drop-out date or study endpoint, whichever came first*/
	else if id_out_date NE '.' then fu_date = id_out_date;
	else fu_date = mdy(12,31,2013);
	if fu_date > mdy(12,31,2013) then fu_date = mdy(12,31,2013); /*if the drop-out date > study endpoint, then the fu_date is set to the study endpoint*/
```

```
fu_time = intck('month', mdy(1,1,1997), fu_date); /*define follow-up time (fu_time) as months being followed from
1997 to follow-up endpoint*/
if fu_time <= 0 then delete; /*delete those already dead before the study*/
run;

proc freq DATA = &ID_list.; /*2*2 table: gender and talc exposure*/
tables id_sex*&depend.  / norow nopercent chisq; RUN;
proc means n mean stderr; /*statistics of age by talc exposure*/ /*statistics of income by talc exposure*/
class &depend.;
var age income;
proc ttest; /*statistics of age by talc exposure, with t-test*/
class &depend.;
var age;
proc ttest; /*statistics of income by talc exposure, with t-test*/
class &depend.;
var income;
run;
proc ttest; /*statistics of follow-up time by talc exposure, with t-test*/
class &depend.;
var fu_time;
run;

/*acquiring the levels of urbanization (urban) from the area code (area_no_i)*/
        /*add trailing zeros to make all strings to have a length of 4, in order to map them to the level of
urbanization*/
        data id_list; set talc.id_list;
        num = lengthn(area_no_i);
        area_no = cats(area_no_i, repeat('0', 4-num));
        area_no = substr(area_no,1,4);
        drop num;        run;
        /*use LEFT JOIN to add "urban" to ID_LIST*/
        PROC SQL; create table id_list as
        select a.*, b.urban
        from ID_list as a left join drug.urban_3_level as b /*dataset "urban_3_level" contains the mapping of level of
urbanization and the area codes*/
        on a.area_no = b.area_no;    QUIT;
        /*go back to talc.ID_LIST and check the frequency*/
        DATA &ID_list.;  SET id_list;
        PROC FREQ; /*2*2 table: level of urbanization and talc exposure*/
        tables urban * &depend. / norow nopercent chisq;
        RUN;

%mend demographic_analysis;
```

CCI calculation.sas

```
/***************************************************************************************************
***********************/
/*Inspired by https://healthcaredelivery.cancer.gov/seermedicare/considerations/charlson.comorbidity.macro.sas
*/
/*Reference:
    Klabunde, C. N., Potosky, A. L., Legler, J. M., & Warren, J. L. (2000). Development of a comorbidity index using
physician claims data. Journal of clinical epidemiology, 53(12), 1258-1267.*/
/***************************************************************************************************
***********************/
/*Calculate the Charlson Comorbidity Index (CCI)*/
/* Calculate the CCI based on icd-9-cm codes (and three visits with the same diagnosis in one year)*/
        /*Prerequisite:
                DRUG.cci (file with the ICD9 codes of the comorbidities)
                D102 or the CD files (datasets of ambulatory care expenditures by visit)
        */
    /* input parameter:
                        outfile: the target file containing the ID list
                ouput: "&outfile._ch" (marking the charlson score on the ID list)
        */

%MACRO dx2times(outfile);
        /*For DRUG.cci (CCI diagnoses and their corresponding icd9):
        output: work.cci*/
        DATA cci; SET DRUG.cci;
        run;

        /*creating macro: Create a 'look up' list for a where statement*/
        %macro comorblist(var);
                %global &var;
                proc sql;
                        select quote(trim(&var.)) into :&var
                        separated by " "
                        from cci
                        where &var. is not missing;
                quit;
        %mend comorblist;
        /*For diagnoses in CCI: Create 'look up' lists for comorbidities*/
        ods select none; /*NOPRINT */
        %comorblist(acute_mi);
        %comorblist(history_mi);
        %comorblist(chf);
        %comorblist(pvd);
        %comorblist(cvd);
```

```
%comorblist(copd);
%comorblist(dementia);
%comorblist(paralysis);
%comorblist(diabetes);
%comorblist(diabetes_comp);
%comorblist(renal_disease);
%comorblist(mild_liver_disease);
%comorblist(liver_disease);
%comorblist(ulcers);
%comorblist(rheum_disease);
%comorblist(aids);
ods select all; /*reopen ODS function*/

/*For m.CD1997 (ambulatory care expenditures by visit):
1. add trailing zeros to the icd9 codes to make length = 5
2. determine the comorbidities based on the icd9cm codes by using the look-up list of CCI
output: dx1time
*/
DATA dx1time;
set
m.cd1997
;
array dxcode{3} icd9_1 icd9_2 icd9_3;
do i= 1 to dim(dxcode);
        if dxcode(i) in :(&acute_mi) then acute_mi= 1;
        if dxcode(i) in :(&history_mi) then history_mi= 1;
        if dxcode(i) in :(&chf) then chf= 1;
        if dxcode(i) in :(&pvd) then pvd= 1;
        if dxcode(i) in :(&cvd) then cvd= 1;
        if dxcode(i) in :(&copd) then copd= 1;
        if dxcode(i) in :(&dementia) then dementia= 1;
        if dxcode(i) in :(&paralysis) then paralysis= 1;
        if dxcode(i) in :(&diabetes) then diabetes= 1;
        if dxcode(i) in :(&diabetes_comp) then diabetes_comp= 1;
        if dxcode(i) in :(&renal_disease) then renal_disease= 1;
        if dxcode(i) in :(&mild_liver_disease) then mild_liver_disease= 1;
        if dxcode(i) in :(&liver_disease) then liver_disease= 1;
        if dxcode(i) in :(&ulcers) then ulcers= 1;
        if dxcode(i) in :(&rheum_disease) then rheum_disease= 1;
        if dxcode(i) in :(&aids) then aids= 1;
end;
drop i; run;

/*calculate the frequency of diagnoses: looking for >= 2 OPD visits*/
```

```
proc means data=dx1time nway noprint;
class id;
var acute_mi--aids;
output out=dx2times  sum = ;
run;


*** Calculate the CCI for prior conditions;
DATA charlson; SET dx2times;
array comorb{16} acute_mi--aids;
do i = 1 to dim(comorb);
        if comorb(i) >= 2 then comorb(i) = 1;  /*diagnosis criteria:  >= 2 OPD visits*/
        else comorb(i) = 0;
end;
drop i;
Charlson = (acute_mi or history_mi) +
      (chf) +
      (pvd) +
      (cvd) +
      (copd) +
      (dementia) +
      (diabetes and not diabetes_comp) +
      (mild_liver_disease and not liver_disease) +
      (ulcers) +
      (rheum_disease) +
      (paralysis*2) +
      (renal_disease*2) +
      (diabetes_comp*2) +
      (liver_disease*3) +
      (aids*6);
RUN;

/*Paste Charlson score back to the outfile*/
PROC SQL; CREATE TABLE &outfile. AS /*outfile: the target file for marking the diagnoses*/
        select a.*, b.charlson
        from &outfile. as a left join charlson as b
        on a.id = b.id;
QUIT;

%mend dx2times;
```

Chinese_herb_process.sas

```
/* Assessing the amount of talc exposure and dealing with the missing value*/
```

```
/*Parameter:
drug_data: dataset with drug prescription data
*/
%macro chinese_herb_process(drug_data);
data  &drug_data.;
set &drug_data.;
if drug_day=0 then drug_day =1;                                    /*if the length of prescription is 0  (drug_day = 0), then change it into drug_day = 1 because it is a STAT use. */
if total_qty/drug_day>20 then total_qty=total_qty/100;     /*If daily dose > 20g, then it makes little clinical sense so we divide the total quantity of prescription (total_qty) by 100, because in the reimbursement process it is a common typing error to mistaken 1.00 as 100 */
proc sort;
by id;
run;
/*mean imputation*/
PROC SQL; CREATE TABLE &drug_data.(drop = x) as
select *, case
        when x = 0 then sum(x) / sum(drug_day) * drug_day /*imputation for total_qty = 0 with average quantity per day multiplied by drug_day*/
        else x
        end as total_qty
FROM talc.exposure_data(rename=(total_qty=x)); /*rename total_qty to x, and then we modify x to a new total_qty; reference: https://communities.sas.com/t5/SAS-Procedures/SQL-how-to-exclude-a-variable-when-doing-select-in-SQL/td-p/91060*/
QUIT;

%mend chinese_herb_process;
```

Summing_exposure.sas

```
/*calculating the cumulative exposure of talc (SUM_dose)*/
        /*Parameters
                ID_list: target file containing the ID list
                drug_data: dataset with drug prescription data
                output_file: the name of the resulting output file
        */
%macro summing_exposure(ID_list, drug_data, output_file);
data exposure_data;         set &drug_data.; /*summing up the exposure by id, multiplied by the weight percentage of talc (talc_frc)*/
by id;
if first.id then do;            SUM_dose = 0;     end;
SUM_dose+total_qty * talc_frc;                    RUN;

/*outputing the cumulative exposure (sum_dose) and cumulative length of prescription (sum_day) for each ID*/
```

```
data exposure_byid (keep=id SUM_dose func_date);
set exposure_data;
by id;
if last.id then output;
run;

/*mapping the exposure data to the target dataset by ID*/
PROC SQL;
create table &output_file. as
select a.*, b.sum_dose, b.func_date
from &ID_list. as a left join exposure_byid as b
on a.id = b.id;
QUIT;

%mend summing_exposure;
```

Level_of_dose.sas

```
/*grouping of the talc exposure into 3 levels*/
        /* Parameters:
                target_file: the target dataset for Cox regression
                scalar: scalar variable to be categorized by the cut-off values
                cutoff01: cut-off point 01
                cutoff02: cut-off point 02
                level: categorical variable resulting from the cut-offs
                depend: dependent variable
        */

%macro level_of_dose(target_file, scalar, cutoff01, cutoff02, level, depend);
PROC SORT data = &target_file.; by id func_date; run;  /*transforming the scalar into the levels*/
data &target_file.; set &target_file.;   by id func_date;
if &scalar. <= &cutoff01. | &scalar. = '.' then &level. = 'low    '; /*low dose: <=cutoff01*/
if &scalar. > &cutoff01. & &scalar. <= &cutoff02. then &level. = 'medium'; /*medium dose: cutoff01~cutoff02*/
if &scalar. > &cutoff02. then &level. = 'high   ';            run; /*high dose: >cutoff02*/

proc freq data = &target_file.; /*2*2 table: the dependent variable and the categorical variable */
tables &depend.*&level. / norow nopercent chisq;          run;

proc summary PRINT N MEAN STD MEDIAN Min Max MEDIAN Q1 Q3; /*check the summary of the scalar
variable*/
var &scalar.;
run;

proc ttest; /*statistics of the scalar variable by the dependent variable, with t-test*/
```

```
class &depend.;
var &scalar.;
run;

%mend level_of_dose;
```

Time_dependent_exposure.sas

```
/*"time-dependent process": We treated the talc exposure as a time-dependent variable
in order to eliminate the immortal time bias, which is a form of selection bias arising
when the period between cohort entry and date of first exposure to a drug is either misclassified
or simply excluded because the event of interest has not occurred*/
/*For patients ever received medical prescription of talc, we considered the time interval
between the beginning of the study (January 1st 1997) and the date of first prescription of talc
to be a non-exposure period, whereas the time interval from the date of first prescription of talc
to the endpoint of follow-up was recognized as an exposure period. */
	/*Parameter
		drug_data: dataset with drug prescription data
	*/

%macro time_dependent_exposure(drug_data);

 /*calculating the end date of prescription (drug_end_date) */
data exposure_data;			set &drug_data.;
format drug_end_date date9.;
drug_end_date = func_date + drug_day;

/*Sorting the prescription data by id and the prescription date*/
proc sort;
by id func_date;
run;

/*Summarizing multiple times of exposure into a single period of exposure:
the time interval from the date of first prescription of talc to the endpoint of follow-up
was recognized as an exposure period*/
	/*new variables:
	time_dependent_func_date: date of first exposure
	sum_day: cumulative length of prescription
	sum_dose: cumulative dose of prescription
	*/
data exposure_data_byid;
set exposure_data;
by id func_date;
format time_dependent_func_date date9.;
```

```
if first.id then do;
		sum_day = 0;
		sum_dose = 0;
		time_dependent_func_date = func_date; /*time_dependent_func_date is the date of first prescription*/
end;
sum_day + drug_day; /*calculating the sum_day*/
sum_dose + total_qty * talc_frc; /*calculating the sum_dose*/
time_dependent_func_date + 0;
func_date = time_dependent_func_date; /*transferring the value of time_dependent_func_date back to func_date, so the func_date is the date of first prescription. Then we dropped time_dependent_func_date*/
drop time_dependent_func_date;
if last.id then output;
drop total_qty drug_day;
run;


/*Identifying the non-exposure period: the time interval between the beginning of the study
(January 1st 1997) and the date of first prescription of talc is a "non-exposure period"*/
		/*new variables:
		no_drug_begin: the start date of the non-exposure period. It will be renamed into func_date.
		no_drug_end: the end date of the non-exposure period.  It will be renamed into drug_end_date.
		*/
data non_exposure_data;
set exposure_data_byid;
format no_drug_end no_drug_begin date9.;
no_drug_end = func_date;	/*the date of prescription is the end date of the non-exposure period*/
no_drug_begin = mdy(1,1,1997); /*the start date of the non-exposure period is the beginning of the study (January 1st 1997)*/
keep id no_drug_begin no_drug_end;
rename no_drug_end = drug_end_date; /*renaming the end date of the non-exposure period into drug_end_date, for subsequent merging of the data of exposure period and the data of non-exposure period*/
rename no_drug_begin = func_date; /*renaming the start date of the non-exposure period into func_date, for subsequent merging of the data of exposure period and the data of non-exposure period*/
run;

/*merging of the data of exposure period and the data of non-exposure period,
and sorting it by the ID and the start date (func_date)*/
data &drug_data.;
set exposure_data_byid non_exposure_data;
if sum_day = . then sum_day = 0;
if sum_dose = . then sum_dose = 0; /*if we see sum_dose = 0, then we know it is a non-exposure period, and vise versa.*/
proc sort;
by id func_date;
```

```
run;

%mend time_dependent_exposure;
```

Before_regression_process.sas

```
/*Procedures before doing the regression*/
        /* Parameters:
        ID_list: target file containing the ID list
        drug_data: dataset with drug prescription data
        output_file: the name of the resulting output file for subsequent regression
        */
%macro before_regression_process(ID_list, drug_data, output_file);

/*Mapping the exposure data to the target cohort (the "id_list")*/
PROC SQL; /*exposed dataset: to be right-joined to drug_data*/
create table before_regression_exposed as
select a.*, b.*
from &ID_list. as a, &drug_data. as b
where a.id = b.id;
QUIT;
PROC SQL; /*non-exposed dataset*/
create table before_regression_nonexposed as
select *
from &ID_list.
where id not in (select id from &drug_data.);
QUIT;
DATA &output_file.; /*all = exposed + non-expossed*/
SET before_regression_exposed before_regression_nonexposed;
drop ever_talc fu_time; /*whether she had ever taken talc (ever_talc) needs to be re-assessed*/     /*follow-up time
(fu_time) needs to be re-assessed*/
RUN;

/*For each time period: set up the event status and the time-to-event*/
PROC SORT data = &output_file.; by id func_date; run;
DATA &output_file.;        set &output_file.;  by id func_date;
        /*set up time-to-event: fu_date, func_date, time (year)*/
        if not last.id then fu_date = drug_end_date; /*if the time period is not the last for that person, set the follow-
up endpoint (fu_date) to the end date of that period (drug_end_date)*/
        if func_date = '.' then func_date = mdy(1,1,1997); /*For people without talc exposure, set up a non-exposure
period from Jan/01/1997 to follow-up end date (fu_date)*/
        time = (fu_date - func_date)/365.25; /*The unit of time-to-event is "year" in the regression*/
        if sum_dose >0 then ever_talc = 1; else ever_talc = 0; /*ever_talc (whether she had ever taken talc) needs to
be rechecked*/
```

```
		fu_time = intck('month', func_date, fu_date); /*fu_time (follow-up time) needs to be re-calculated*/
		if ca = '.' then ca = 0;
		if last.id & ca = 1 then ca = 1; else ca = 0; /*if the time period is not the last for that person, then the event
has not happened (event = 0) */
		run;

/*defining "elder" as age >= 65*/
DATA &output_file.;		set &output_file.;
if age >= 65 then elder = 1; else elder = 0; run;

%mend before_regression_process;
```

Num_of_event_table.sas

```
/*Reporting numbers of outcome events or summary measures over time.*/
	/*Parameters
		ID_list: target file containing the ID list to calculate the of outcome events
		event: the name of variable of interest (the event)
		fu_time_year: the variable name containing the follow-up time (year)
	*/
%macro num_of_event_table(ID_list);
DATA ID_list; SET &ID_list.;
if age >= 65 then elder = 1; else elder = 0;
fu_time_year = fu_time / 12;

/*grouping variables: age, gender, levels of urbanization,
co-morbidities, ever exposed to talc, levels of talc exposure*/
PROC tabulate  data = ID_LIST;
class elder id_sex urban CCI ever_talc level_dose/missing;
var ca fu_time_year;
table elder * ca , sum;
table elder * fu_time_year , sum;
table id_sex * ca , sum;
table id_sex * fu_time_year , sum;
table urban * ca , sum;
table urban * fu_time_year , sum;
table CCI * ca , sum;
table CCI * fu_time_year , sum;
table ever_talc * ca , sum;
table ever_talc * fu_time_year , sum;
table level_dose * ca , sum;
table level_dose * fu_time_year , sum;
RUN;
%mend num_of_event_table;
```

Cox_regression_of_talc.sas

```
/*2018.01 Cox regression of talc
dependent variable: diagnosis of stomach cancer (ca)
explanatory variables: ever exposure to talc (ever_talc), level of talc exposure (level_dose)
confounders: age, gender, CCI*/
	/* Parameter:
		ID_list: target file containing the time, the outcome, and the variables.
	*/
data Inrisks_ever_talc; /*this dataset would be used as the baseline statement of
the PROC PHREG (Cox regression on ever exposure to talc) for estimating the survival function*/
  input ever_talc Id $;
  datalines;
0 unexposed
1 exposed
;
data Inrisks_level_dose; /*this dataset would be used in the baseline statement of
the PROC PHREG (Cox regression on level of talc exposure) for estimating the survival function*/
  input level_dose $ Id $;
  datalines;
medium medium_e
high high_e
low low_e
;


%macro Cox_regression_of_talc(ID_list);
	/*Cox regression (on ever exposure to talc): crude HR*/
	proc phreg data = &ID_list. ;
	class ever_talc (ref = '0') / param = ref;
	model time*ca(0)= ever_talc;
	baseline covariates=Inrisks_ever_talc out=Pred1 survival=_all_ / rowid=Id; /*calculate absolute risk by the
estimated survival function*/
	run;
		/*print out the 10-y absolute risks*/
		proc print data=Pred1(where=(ever_talc = 1 and round(time,.01)=10)); /*10-y absolute risks for
exposure group */
		proc print data=Pred1(where=(ever_talc = 0 and round(time,.01)=10)); /*10-y absolute risks for
unexposed group */
		run;
		/*Wilcoxin test for survival analysis*/
		PROC LIFETEST DATA= &ID_LIST. NOTABLE OUTSURV= surv1;
		TIME time * ca(0);
		STRATA ever_talc;
		RUN;
```

```
/*Survival plot*/
proc sgplot data=surv1;
  step x=time y=survival / group=ever_talc lineattrs=(pattern=solid) name='s';
  keylegend 's';
  yaxis min=0.99;
run;


/*Cox regression (on level of talc exposure): crude HR*/
proc phreg data = &ID_list.;
class level_dose (ref = 'low    ') / param = ref;
model time*ca(0)= level_dose;
baseline covariates=Inrisks_level_dose out=Pred2 survival=_all_ / rowid=Id; /*calculate absolute risk by the estimated survival function*/
run;
/*print out the 10-y absolute risks*/
proc print data=Pred2(where=(level_dose = 'low    ' and round(time,.01)=10)); /*10-y absolute risks for low exposure group (non-elder, female, low CCI)*/
proc print data=Pred2(where=(level_dose = 'medium' and round(time,.01)=10)); /*10-y absolute risks for medium exposure group (non-elder, female, low CCI)*/
proc print data=Pred2(where=(level_dose = 'high   ' and round(time,.01)=10)); /*10-y absolute risks for high exposure group (non-elder, female, low CCI)*/
run;
/*Wilcoxin test for survival analysis*/
PROC LIFETEST DATA= &ID_LIST. NOTABLE OUTSURV= surv2;
TIME time * ca(0);
STRATA level_dose;
RUN;
/*Survival plot*/
proc sgplot data=surv2;
  step x=time y=survival / group=level_dose lineattrs=(pattern=solid) name='s';
  keylegend 's';
  yaxis min=0.99;
run;

/*Cox regression (on ever exposure to talc): adjusted HR*/
proc phreg data = &ID_list.;
class elder (ref = '0') / param = ref; /*confounders: age, gender, CCI*/
class id_sex (ref = 'F') / param = ref;
class CCI (ref = 'low ') / param = ref;
class ever_talc (ref = '0') / param = ref;
model time*ca(0)= elder id_sex CCI ever_talc;
run;
```

```
/*Cox regression (on level of talc exposure): adjusted HR*/
        proc phreg data = &ID_list. ;
        class elder (ref = '0') / param = ref; /*confounders: age, gender, CCI*/
        class id_sex (ref = 'F') / param = ref;
        class CCI (ref = 'low ') / param = ref;
        class level_dose (ref = 'low    ') / param = ref;
        model time*ca(0)= elder id_sex CCI level_dose;
        run;
%mend Cox_regression_of_talc;
```

Appendix 3. Drug codes of Chinese herbal medicine containing talc

Filename: talc_chinese.sas7bdat

Description: This dataset comprises all drug codes of Chinese herbal medicine containing talc.

Coding of variables:

- Drug_no: Pharmaceutical codes by the National Health Insurance Administration (NHIA)
- Drug_name: Name of drug (in Chinese)

  滑石 = Talc powder

  六一散 = Six-to-One Powder (6:1 ratio between talc and licorice)
- Talc_frc: Weight fraction of talc

drug_no	drug_name	talc_frc
A050285	滑石	1
A050300	滑石	1
A050442	滑石	1
A050508	滑石	1
A050520	滑石	1
A050585	滑石	1
A050735	滑石	1
A051442	滑石	1
A052631	滑石	1
A052711	滑石	1
A053363	滑石	1
A054129	滑石	1
A054338	滑石	1
A054578	滑石	1
A054747	滑石	1
A054846	滑石	1
A055747	滑石	1
A056813	滑石	1
A001265	滑石	1

A051599	滑石	1
A053634	滑石	1
B001729	六一散	0.8571
B012253	六一散	0.8571
B012940	六一散	0.8571
B013404	六一散	0.8571
A031913	六一散	0.8571
A032699	六一散	0.8571
A035552	六一散	0.8571
A038080	六一散	0.8571

Appendix 4. Level of urbanization according to the region code in Taiwan

Filename: urban_3_level.sas7bdat

Description: This dataset comprises region codes of the residential region and corresponding levels of urbanization according to previous publication, with level 1 referring to the "most urbanized" and level 3 referring to the "least urbanized" communities.

Coding of variables:

- Dist_name: Name of the residential region (in Chinese)
- REG_ZIP_CODE: Postal code
- Area_no: Region code
- Urban: Level of urbanization

Reference:

Liu, C.-Y., et al., Incorporating development stratification of Taiwan townships into sampling design of large scale health interview survey. J Health Manag, 2006. 4(1): p. 1-22.

Dist_Name	REG_ZIP_CODE	Urban	area_no
台北市	.	high	100
台北市松山區	105	high	101
台北市大安區	106	high	102
台北市大同區	103	high	109
台北市中山區	104	high	110
台北市內湖區	114	medium	111
台北市南港區	115	medium	112
台北市士林區	111	medium	115
台北市北投區	112	medium	116
台北市信義區	110	high	117
台北市中正區	100	high	118
台北市萬華區	108	high	119
台北市文山區	116	medium	120
高雄市	.	high	200

高雄市鹽埕區		803 high	201
高雄市鼓山區		804 medium	202
高雄市左營區		813 medium	203
高雄市楠梓區		811 medium	204
高雄市三民區		807 low	205
高雄市新興區		800 high	206
高雄市前金區		801 high	207
高雄市苓雅區		802 high	208
高雄市前鎮區		806 medium	209
高雄市旗津區		805 high	210
高雄市小港區		812 medium	211
基隆市	.	medium	1100
基隆市中正區		202 medium	1101
基隆市七堵區		206 medium	1102
基隆市暖暖區		205 medium	1103
基隆市仁愛區		200 medium	1104
基隆市中山區		203 medium	1105
基隆市安樂區		204 medium	1106
基隆市信義區		201 medium	1107
新竹市	.	medium	1200
新竹市東區		300 medium	1201
新竹市北區		300 medium	1204
新竹市香山區		300 medium	1205
台中市	.	high	1700
台中市中區		400 high	1701
台中市東區		401 medium	1702
台中市西區		403 high	1703
台中市南區		402 high	1704
台中市北區		404 high	1705
台中市西屯區		407 medium	1706
台中市南屯區		408 medium	1707
台中市北屯區		406 medium	1708
台南市	.	medium	2100
台南市東區		701 high	2101
台南市南區		702 medium	2102
台南市西區	.	medium	2103
台南市北區		704 medium	2104
台南市中區	.	high	2105

台南市安南區	709 medium	2106
台南市安平區	708 medium	2107
台南市中西區	534 high	2108
嘉義市	. medium	2200
嘉義市東區	600 medium	2201
嘉義市西區	600 medium	2202
台北縣	. low	3100
台北縣板橋市	220 high	3101
台北縣三重市	241 high	3102
台北縣永和市	234 high	3103
台北縣中和市	235 high	3104
台北縣新店市	231 medium	3105
台北縣新莊市	242 high	3106
台北縣樹林鎮	238 medium	3107
台北縣鶯歌鎮	239 medium	3108
台北縣三峽鎮	237 low	3109
台北縣淡水鎮	251 medium	3110
台北縣汐止鎮	221 medium	3111
台北縣瑞芳鎮	224 low	3112
台北縣土城鄉	236 low	3113
台北縣蘆洲鄉	247 low	3114
台北縣五股鄉	248 medium	3115
台北縣泰山鄉	243 medium	3116
台北縣林口鄉	244 medium	3117
台北縣深坑鄉	222 medium	3118
台北縣石碇鄉	223 low	3119
台北縣坪林鄉	232 low	3120
台北縣三芝鄉	252 low	3121
台北縣石門鄉	253 low	3122
台北縣八里鄉	249 medium	3123
台北縣平溪鄉	226 low	3124
台北縣雙溪鄉	227 low	3125
台北縣貢寮鄉	228 low	3126
台北縣金山鄉	208 low	3127
台北縣萬里鄉	207 low	3128
台北縣烏來鄉	233 low	3129
桃園縣	. medium	3200
桃園縣桃園市	330 medium	3201

桃園縣中壢市	320 medium	3202
桃園縣大溪鎮	335 low	3203
桃園縣楊梅鎮	326 medium	3204
桃園縣蘆竹鄉	338 medium	3205
桃園縣大園鄉	337 medium	3206
桃園縣龜山鄉	333 high	3207
桃園縣八德鄉	334 low	3208
桃園縣龍潭鄉	325 medium	3209
桃園縣平鎮鄉	324 low	3210
桃園縣新屋鄉	327 low	3211
桃園縣觀音鄉	328 low	3212
桃園縣復興鄉	336 low	3213
新竹縣 .	low	3300
新竹縣關西鎮	306 low	3301
新竹縣新埔鎮	305 low	3302
新竹縣竹東鎮	310 medium	3303
新竹縣竹北市	302 medium	3305
新竹縣湖口鄉	303 medium	3306
新竹縣橫山鄉	312 low	3307
新竹縣新豐鄉	304 medium	3308
新竹縣芎林鄉	307 low	3309
新竹縣寶山鄉	308 low	3310
新竹縣北埔鄉	314 low	3311
新竹縣峨眉鄉	315 low	3312
新竹縣尖石鄉	313 low	3313
新竹縣五峰鄉	311 low	3314
宜蘭縣 .	low	3400
宜蘭縣宜蘭市	260 medium	3401
宜蘭縣羅東鎮	265 medium	3402
宜蘭縣蘇澳鎮	270 low	3403
宜蘭縣頭城鎮	261 low	3404
宜蘭縣礁溪鄉	262 low	3405
宜蘭縣壯圍鄉	263 low	3406
宜蘭縣員山鄉	264 low	3407
宜蘭縣冬山鄉	269 low	3408
宜蘭縣五結鄉	268 low	3409
宜蘭縣三星鄉	266 low	3410
宜蘭縣大同鄉	267 low	3411

宜蘭縣南澳鄉	272 low	3412
苗栗縣 .	low	3500
苗栗縣苗栗市	360 medium	3501
苗栗縣苑裡鎮	358 low	3502
苗栗縣通霄鎮	357 low	3503
苗栗縣竹南鎮	350 medium	3504
苗栗縣頭份鎮	351 low	3505
苗栗縣後龍鎮	356 low	3506
苗栗縣卓蘭鎮	369 low	3507
苗栗縣大湖鄉	364 low	3508
苗栗縣公館鄉	363 low	3509
苗栗縣銅鑼鄉	366 low	3510
苗栗縣南庄鄉	353 low	3511
苗栗縣頭屋鄉	362 low	3512
苗栗縣三義鄉	367 low	3513
苗栗縣西湖鄉	368 low	3514
苗栗縣造橋鄉	361 low	3515
苗栗縣三灣鄉	352 low	3516
苗栗縣獅潭鄉	354 low	3517
苗栗縣泰安鄉	365 low	3518
台中縣 .	low	3600
台中縣豐原市	420 low	3601
台中縣東勢鎮	423 low	3602
台中縣大甲鎮	437 low	3603
台中縣清水鎮	436 medium	3604
台中縣沙鹿鎮	433 medium	3605
台中縣梧棲鎮	435 medium	3606
台中縣后里鄉	421 low	3607
台中縣神岡鄉	429 medium	3608
台中縣潭子鄉	427 medium	3609
台中縣大雅鄉	428 medium	3610
台中縣新社鄉	426 low	3611
台中縣石岡鄉	422 low	3612
台中縣外埔鄉	438 low	3613
台中縣大安鄉	439 low	3614
台中縣烏日鄉	414 medium	3615
台中縣大肚鄉	432 medium	3616
台中縣龍井鄉	434 medium	3617

台中縣霧峰鄉	413 medium	3618
台中縣太平鄉	411 low	3619
台中縣大里鄉	412 low	3620
台中縣和平鄉	424 low	3621
彰化縣 .	low	3700
彰化縣彰化市	500 medium	3701
彰化縣鹿港鎮	505 medium	3702
彰化縣和美鎮	508 medium	3703
彰化縣北斗鎮	521 low	3704
彰化縣員林鎮	510 low	3705
彰化縣溪湖鎮	514 low	3706
彰化縣田中鎮	520 low	3707
彰化縣二林鎮	526 low	3708
彰化縣線西鄉	507 low	3709
彰化縣伸港鄉	509 medium	3710
彰化縣福興鄉	506 low	3711
彰化縣秀水鄉	504 low	3712
彰化縣花壇鄉	503 medium	3713
彰化縣芬園鄉	502 low	3714
彰化縣大村鄉	515 medium	3715
彰化縣埔鹽鄉	516 low	3716
彰化縣埔心鄉	513 low	3717
彰化縣永靖鄉	512 low	3718
彰化縣社頭鄉	511 low	3719
彰化縣二水鄉	530 low	3720
彰化縣田尾鄉	522 low	3721
彰化縣埤頭鄉	523 low	3722
彰化縣芳苑鄉	528 low	3723
彰化縣大城鄉	527 low	3724
彰化縣竹塘鄉	525 low	3725
彰化縣溪州鄉	524 low	3726
南投縣 .	low	3800
南投縣南投市	540 low	3801
南投縣埔里鎮	545 low	3802
南投縣草屯鎮	542 low	3803
南投縣竹山鎮	557 low	3804
南投縣集集鎮	552 low	3805
南投縣名間鄉	551 low	3806

南投縣鹿谷鄉	558 low	3807
南投縣中寮鄉	541 low	3808
南投縣魚池鄉	555 low	3809
南投縣國姓鄉	544 low	3810
南投縣水里鄉	553 low	3811
南投縣信義鄉	556 low	3812
南投縣仁愛鄉	546 low	3813
雲林縣 .	low	3900
雲林縣斗六市	640 medium	3901
雲林縣斗南鎮	630 low	3902
雲林縣虎尾鎮	632 low	3903
雲林縣西螺鎮	648 low	3904
雲林縣土庫鎮	633 low	3905
雲林縣北港鎮	651 low	3906
雲林縣古坑鄉	646 low	3907
雲林縣大埤鄉	631 low	3908
雲林縣莿桐鄉	647 low	3909
雲林縣林內鄉	643 low	3910
雲林縣二崙鄉	649 low	3911
雲林縣崙背鄉	637 low	3912
雲林縣麥寮鄉	638 low	3913
雲林縣東勢鄉	635 low	3914
雲林縣褒忠鄉	634 low	3915
雲林縣台西鄉	636 low	3916
雲林縣元長鄉	655 low	3917
雲林縣四湖鄉	654 low	3918
雲林縣口湖鄉	653 low	3919
雲林縣水林鄉	652 low	3920
嘉義縣 .	low	4000
嘉義縣朴子鎮	613 low	4001
嘉義縣布袋鎮	625 low	4002
嘉義縣大林鎮	622 low	4003
嘉義縣民雄鄉	621 medium	4004
嘉義縣溪口鄉	623 low	4005
嘉義縣新港鄉	616 low	4006
嘉義縣六腳鄉	615 low	4007
嘉義縣東石鄉	614 low	4008
嘉義縣義竹鄉	624 low	4009

嘉義縣鹿草鄉	611 low	4010
嘉義縣太保鄉	612 low	4011
嘉義縣水上鄉	608 low	4012
嘉義縣中埔鄉	606 low	4013
嘉義縣竹崎鄉	604 low	4014
嘉義縣梅山鄉	603 low	4015
嘉義縣番路鄉	602 low	4016
嘉義縣大埔鄉	607 low	4017
嘉義縣阿里山	605 low	4018
台南縣 .	low	4100
台南縣新營市	730 medium	4101
台南縣鹽水鎮	737 low	4102
台南縣白河鎮	732 low	4103
台南縣麻豆鎮	721 low	4104
台南縣佳里鎮	722 low	4105
台南縣新化鎮	712 low	4106
台南縣善化鎮	741 low	4107
台南縣學甲鎮	726 low	4108
台南縣柳營鄉	736 low	4109
台南縣後壁鄉	731 low	4110
台南縣東山鄉	733 low	4111
台南縣下營鄉	735 low	4112
台南縣六甲鄉	734 low	4113
台南縣官田鄉	720 low	4114
台南縣大內鄉	742 low	4115
台南縣西港鄉	723 low	4116
台南縣七股鄉	724 low	4117
台南縣將軍鄉	725 low	4118
台南縣北門鄉	727 low	4119
台南縣新市鄉	744 low	4120
台南縣安定鄉	745 low	4121
台南縣山上鄉	743 low	4122
台南縣玉井鄉	714 low	4123
台南縣楠西鄉	715 low	4124
台南縣南化鄉	716 low	4125
台南縣左鎮鄉	713 low	4126
台南縣仁德鄉	717 low	4127
台南縣歸仁鄉	711 medium	4128

台南縣關廟鄉		718 low	4129
台南縣龍崎鄉		719 low	4130
台南縣永康鄉		710 low	4131
高雄縣	.	medium	4200
高雄縣鳳山市		830 medium	4201
高雄縣岡山鎮		820 medium	4202
高雄縣旗山鎮		842 low	4203
高雄縣美濃鎮		843 low	4204
高雄縣林園鄉		832 medium	4205
高雄縣大寮鄉		831 medium	4206
高雄縣大樹鄉		840 medium	4207
高雄縣仁武鄉		814 medium	4208
高雄縣大社鄉		815 medium	4209
高雄縣鳥松鄉		833 high	4210
高雄縣橋頭鄉		825 medium	4211
高雄縣燕巢鄉		824 low	4212
高雄縣田寮鄉		823 low	4213
高雄縣阿蓮鄉		822 low	4214
高雄縣路竹鄉		821 medium	4215
高雄縣湖內鄉		829 medium	4216
高雄縣茄萣鄉		852 medium	4217
高雄縣永安鄉		828 low	4218
高雄縣彌陀鄉		827 medium	4219
高雄縣梓官鄉		826 medium	4220
高雄縣六龜鄉		844 low	4221
高雄縣甲仙鄉		847 low	4222
高雄縣杉林鄉		846 low	4223
高雄縣內門鄉		845 low	4224
高雄縣茂林鄉		851 low	4225
高雄縣桃源鄉		848 low	4226
高雄縣那瑪夏		849 low	4227
屏東縣	.	low	4300
屏東縣屏東市		900 medium	4301
屏東縣潮州鎮		920 low	4302
屏東縣東港鎮		928 low	4303
屏東縣恆春鎮		946 low	4304
屏東縣萬丹鄉		913 low	4305
屏東縣長治鄉		908 low	4306

屏東縣麟洛鄉	909 low	4307
屏東縣九如鄉	904 low	4308
屏東縣里港鄉	905 low	4309
屏東縣鹽埔鄉	907 low	4310
屏東縣高樹鄉	906 low	4311
屏東縣萬巒鄉	923 low	4312
屏東縣內埔鄉	912 low	4313
屏東縣竹田鄉	911 low	4314
屏東縣新埤鄉	925 low	4315
屏東縣枋寮鄉	940 low	4316
屏東縣新園鄉	932 low	4317
屏東縣崁頂鄉	924 low	4318
屏東縣林邊鄉	927 low	4319
屏東縣南州鄉	926 low	4320
屏東縣佳冬鄉	931 low	4321
屏東縣琉球鄉	929 low	4322
屏東縣車城鄉	944 low	4323
屏東縣滿州鄉	947 low	4324
屏東縣枋山鄉	941 low	4325
屏東縣三地門	901 low	4326
屏東縣霧台鄉	902 low	4327
屏東縣瑪家鄉	903 low	4328
屏東縣泰武鄉	921 low	4329
屏東縣來義鄉	922 low	4330
屏東縣春日鄉	942 low	4331
屏東縣獅子鄉	943 low	4332
屏東縣牡丹鄉	945 low	4333
澎湖縣	. low	4400
澎湖縣馬公市	880 low	4401
澎湖縣湖西鎮	885 low	4402
澎湖縣白沙鄉	884 low	4403
澎湖縣西嶼鄉	881 low	4404
澎湖縣望安鄉	882 low	4405
澎湖縣七美鄉	883 low	4406
花蓮縣	. low	4500
花蓮縣花蓮市	970 medium	4501
花蓮縣鳳林鎮	975 low	4502
花蓮縣玉里鎮	981 low	4503

花蓮縣新城鄉	971 medium	4504
花蓮縣吉安鄉	973 medium	4505
花蓮縣壽豐鄉	974 low	4506
花蓮縣光復鄉	976 low	4507
花蓮縣豐濱鄉	977 low	4508
花蓮縣瑞穗鄉	978 low	4509
花蓮縣富里鄉	983 low	4510
花蓮縣秀林鄉	972 low	4511
花蓮縣萬榮鄉	979 low	4512
花蓮縣卓溪鄉	982 low	4513
台東縣 .	low	4600
台東縣台東市	950 low	4601
台東縣成功鎮	961 low	4602
台東縣關山鎮	956 low	4603

Appendix 5. Image files at 20,000x of mineral analysis of talc particles in Taiwan

Description: The sampled talcum powder (License No. 050285) was manufactured from Sun Ten Pharmaceutical Co., Ltd. The image files at 20,000x of mineral analysis of talc were taken on March 30th 2018, using the scanning electron microscopy (SEM) combined with energy dispersive x-ray spectroscopy (EDS). Following the ISO-14966 method, we examined 100 particles and did not detect asbestos or asbestiform fibers.

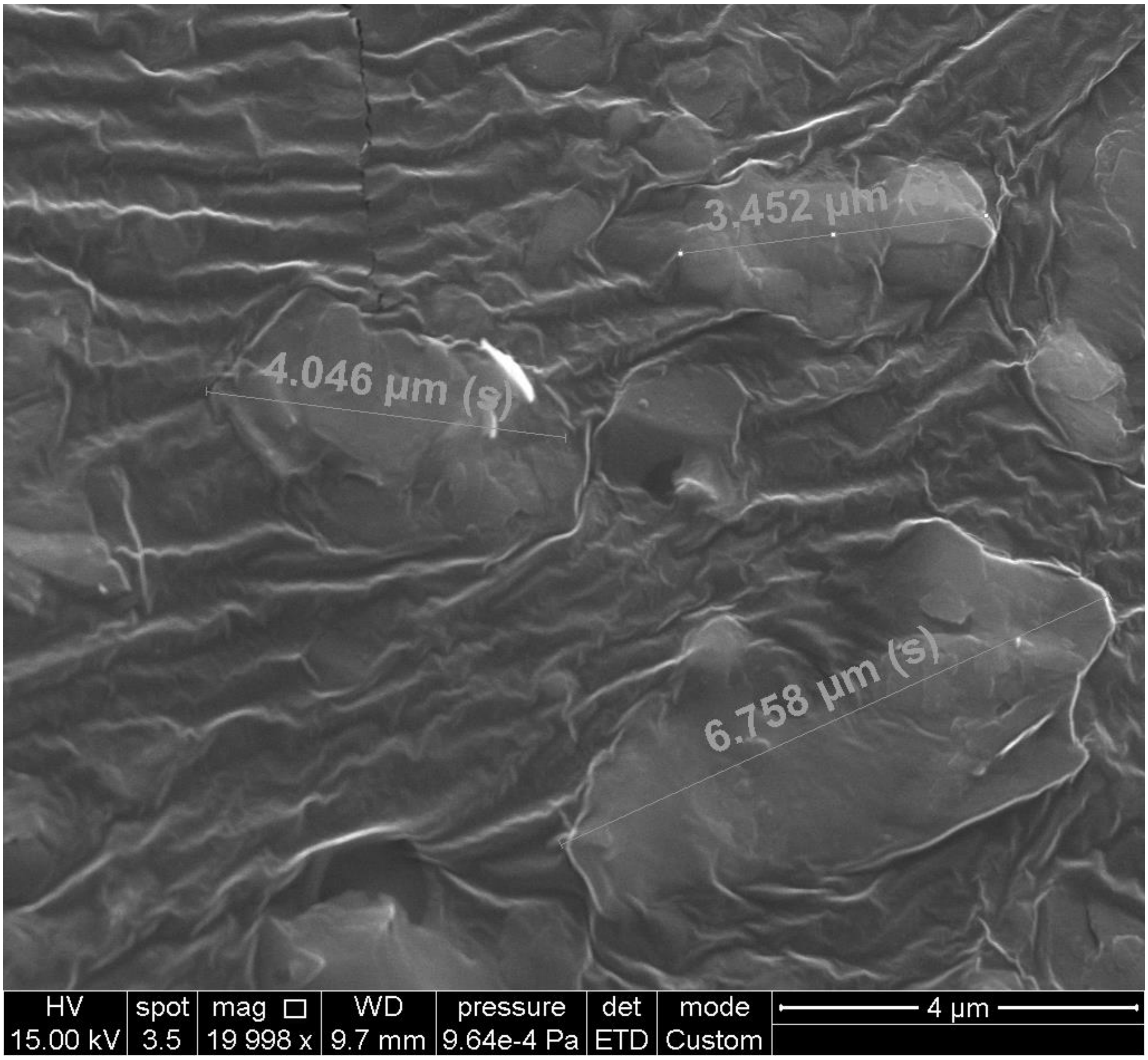

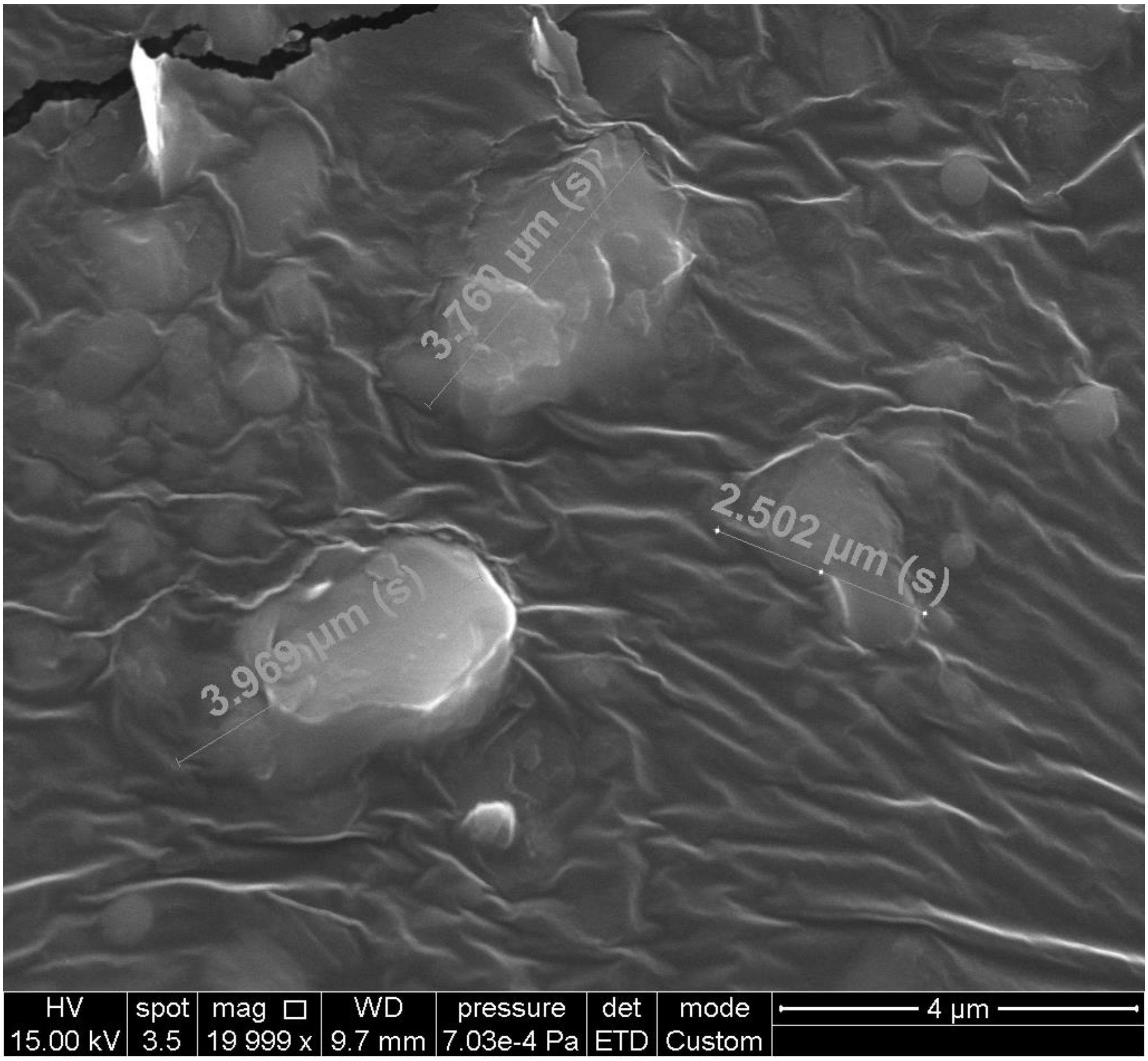
3.760 µm (s)
2.502 µm (s)
3.969 µm (s)
HV
15.00 kV
spot
3.5
mag
19 999 x
WD
9.7 mm
pressure
7.03e-4 Pa
det
ETD
mode
Custom
4 µm

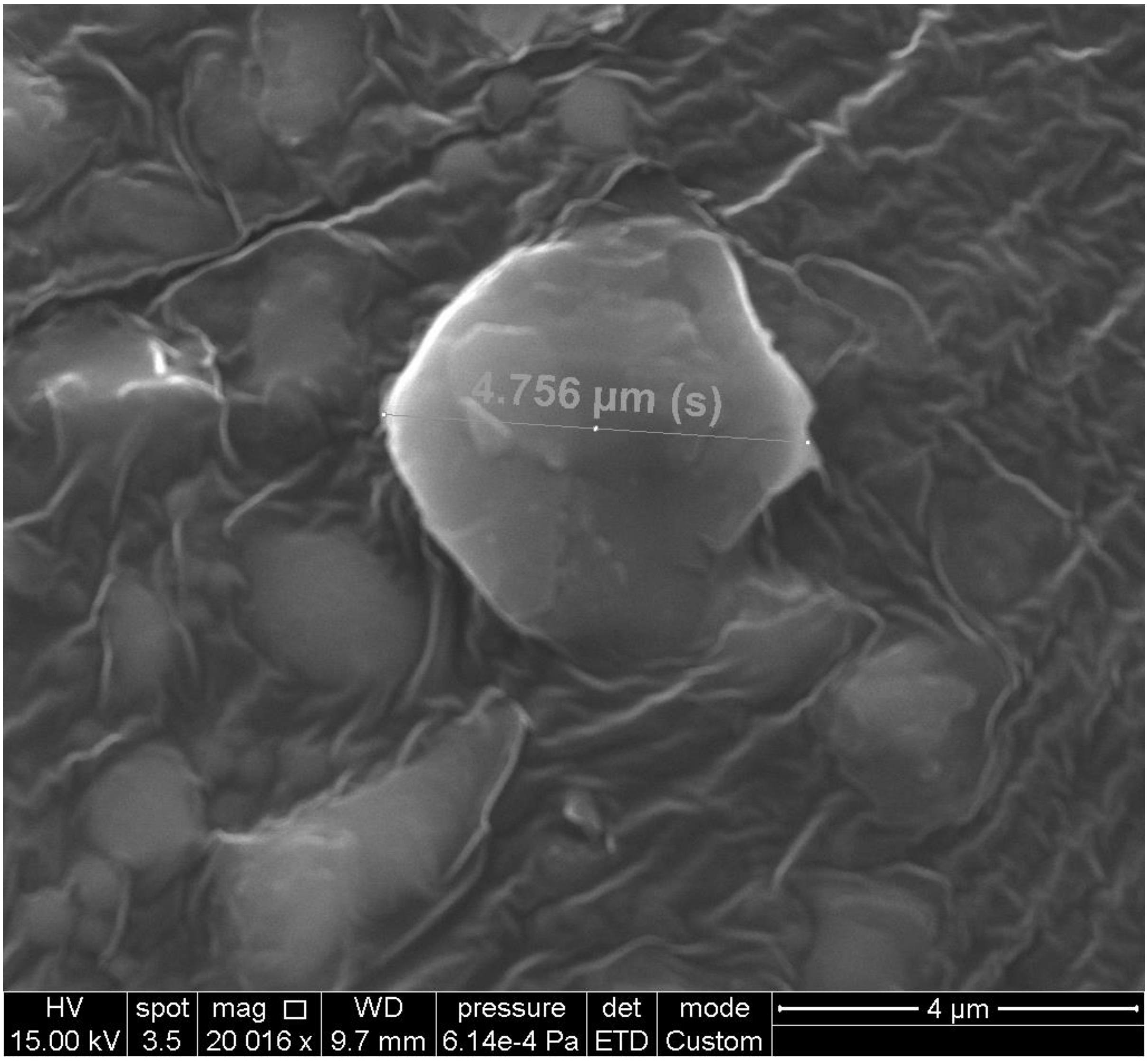
4.756 µm (s)
HV
15.00 kV
spot
3.5
mag
20 016 x
WD
9.7 mm
pressure
6.14e-4 Pa
det
ETD
mode
Custom
4 µm

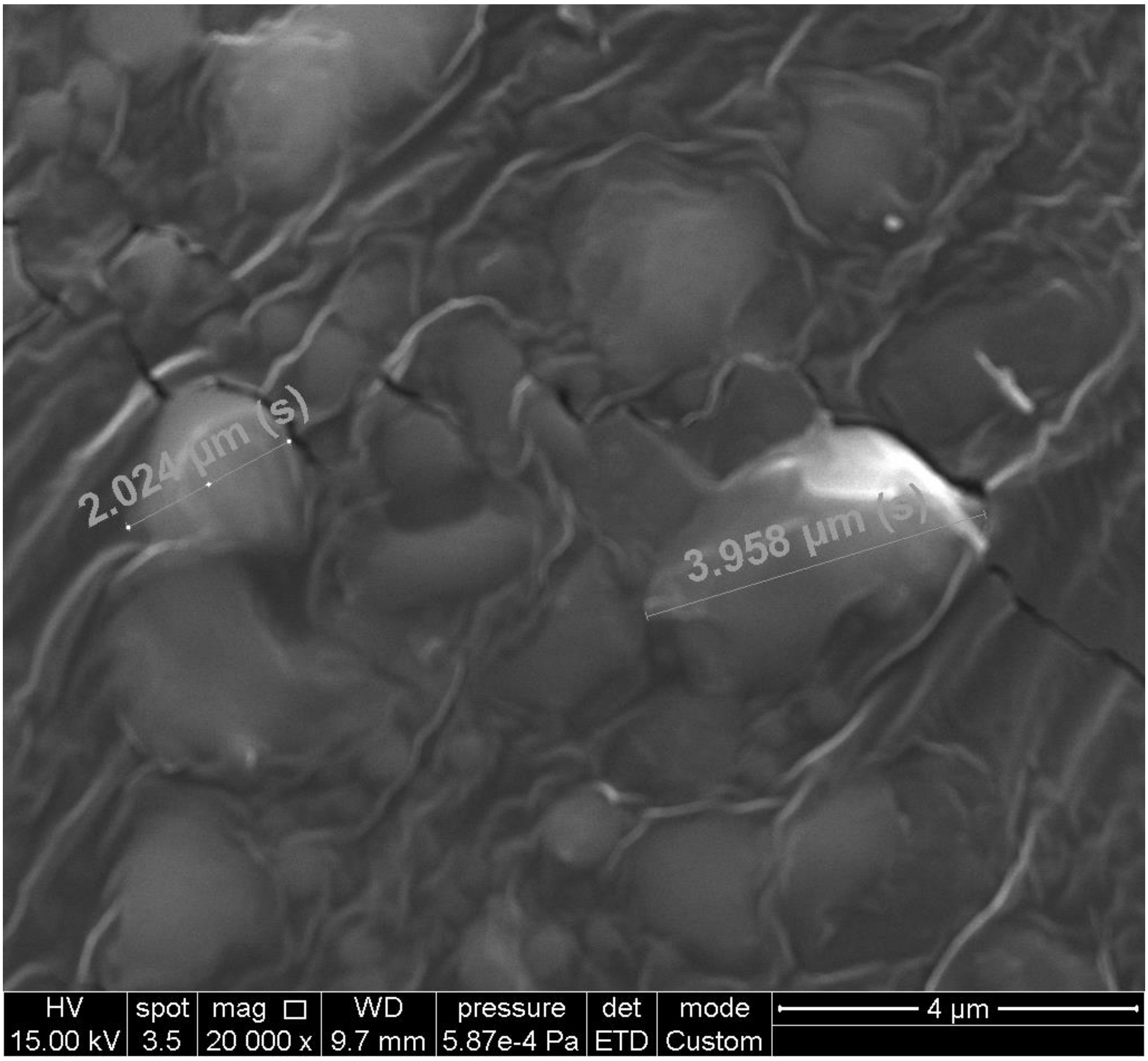
2.024 µm (s)
3.958 µm (s)
HV 15.00 kV
spot 3.5
mag 20 000 x
WD 9.7 mm
pressure 5.87e-4 Pa
det ETD
mode Custom
4 µm

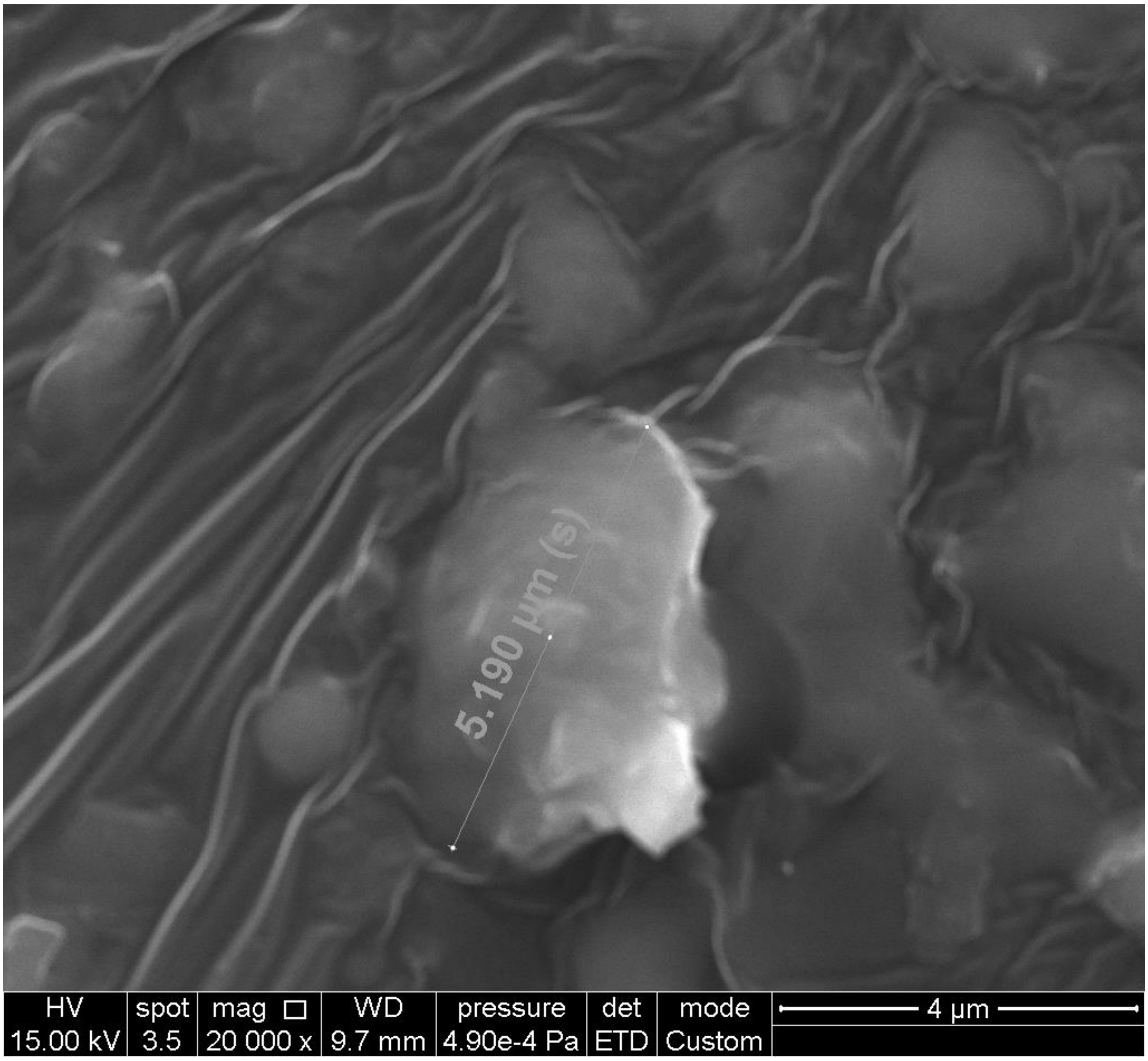
5.190 µm (s)
HV
15.00 kV
spot
3.5
mag
20 000 x
WD
9.7 mm
pressure
4.90e-4 Pa
det
ETD
mode
Custom
4 µm

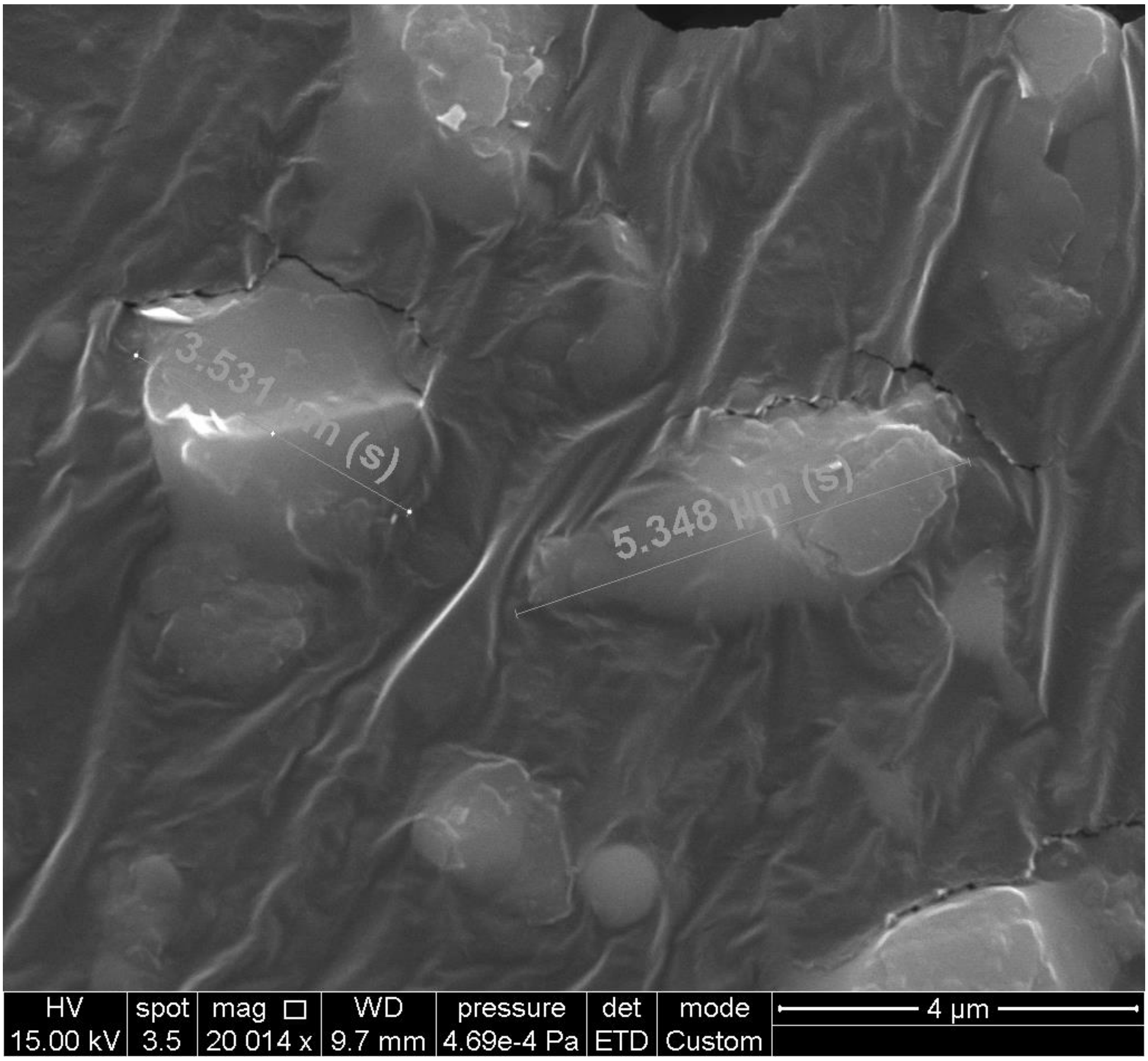
3.531 µm (s)
5.348 µm (s)
HV
15.00 kV
spot
3.5
mag
20 014 x
WD
9.7 mm
pressure
4.69e-4 Pa
det
ETD
mode
Custom
4 µm

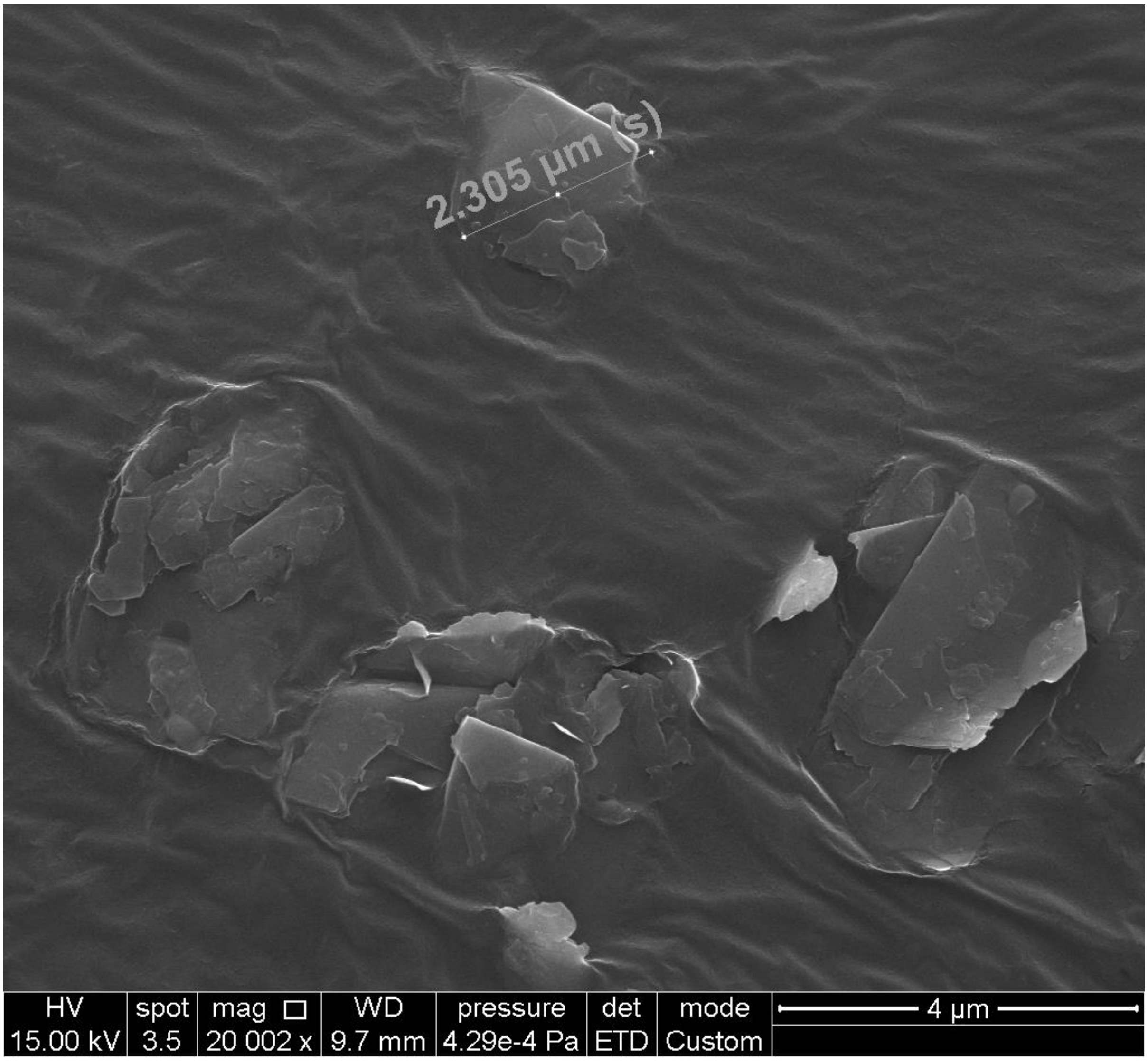
2.305 µm (s)
HV
15.00 kV
spot
3.5
mag
20 002 x
WD
9.7 mm
pressure
4.29e-4 Pa
det
ETD
mode
Custom
4 µm

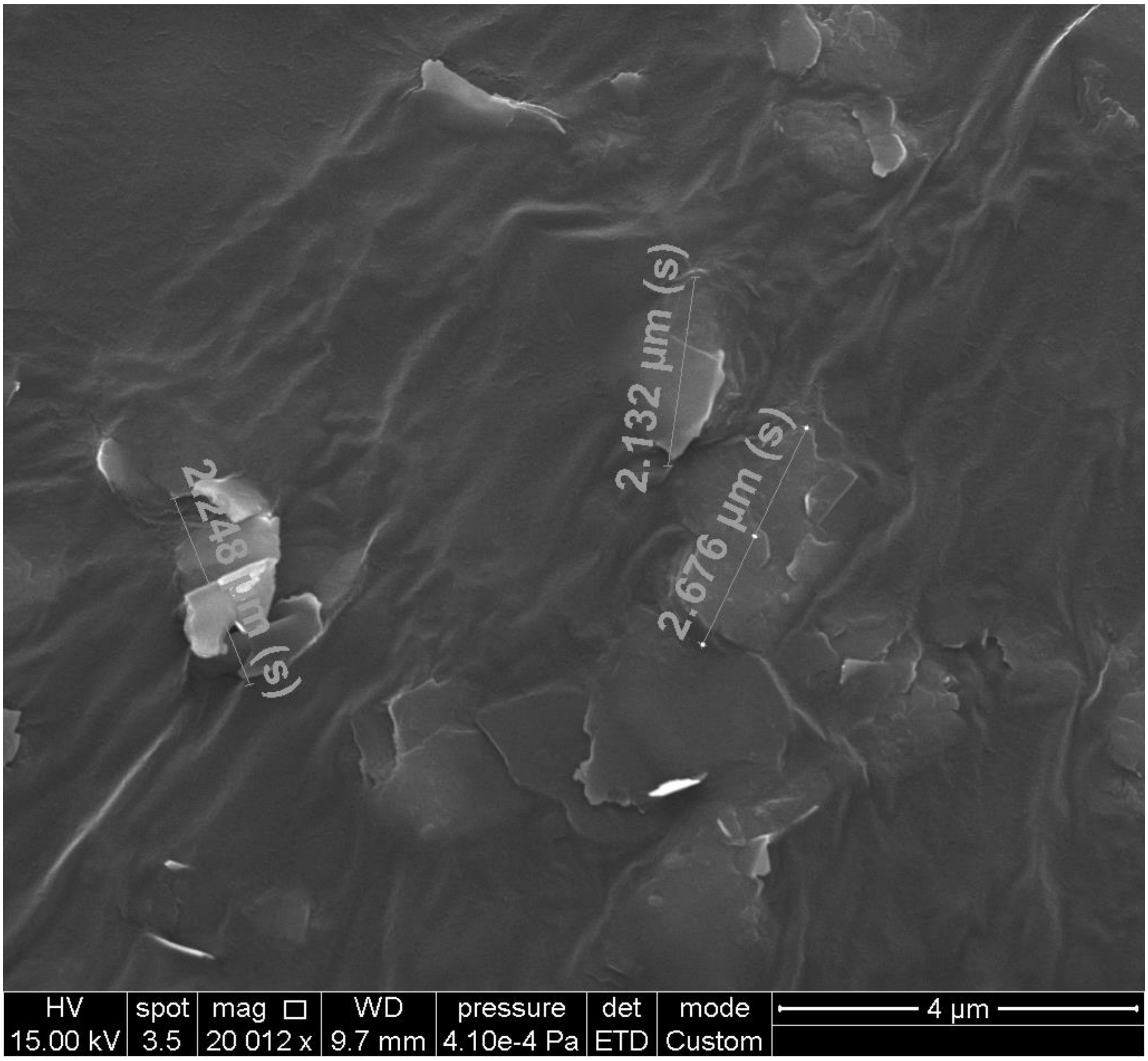
2.132 µm (s)
2.676 µm (s)
HV 15.00 kV
spot 3.5
mag 20 012 x
WD 9.7 mm
pressure 4.10e-4 Pa
det ETD
mode Custom
4 µm

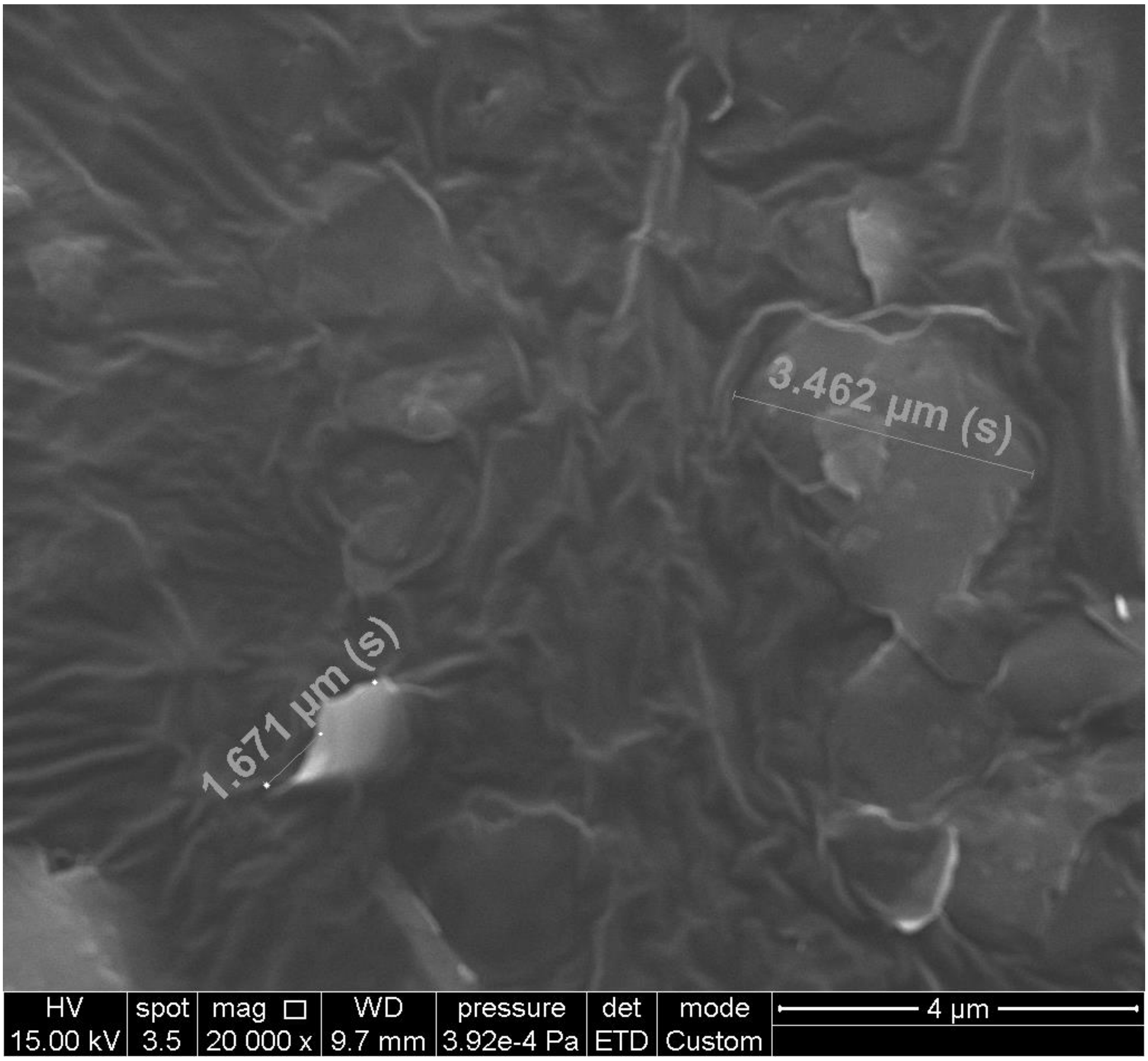

3.462 µm (s)
1.671 µm (s)
HV
15.00 kV
spot
3.5
mag
20 000 x
WD
9.7 mm
pressure
3.92e-4 Pa
det
ETD
mode
Custom
4 µm

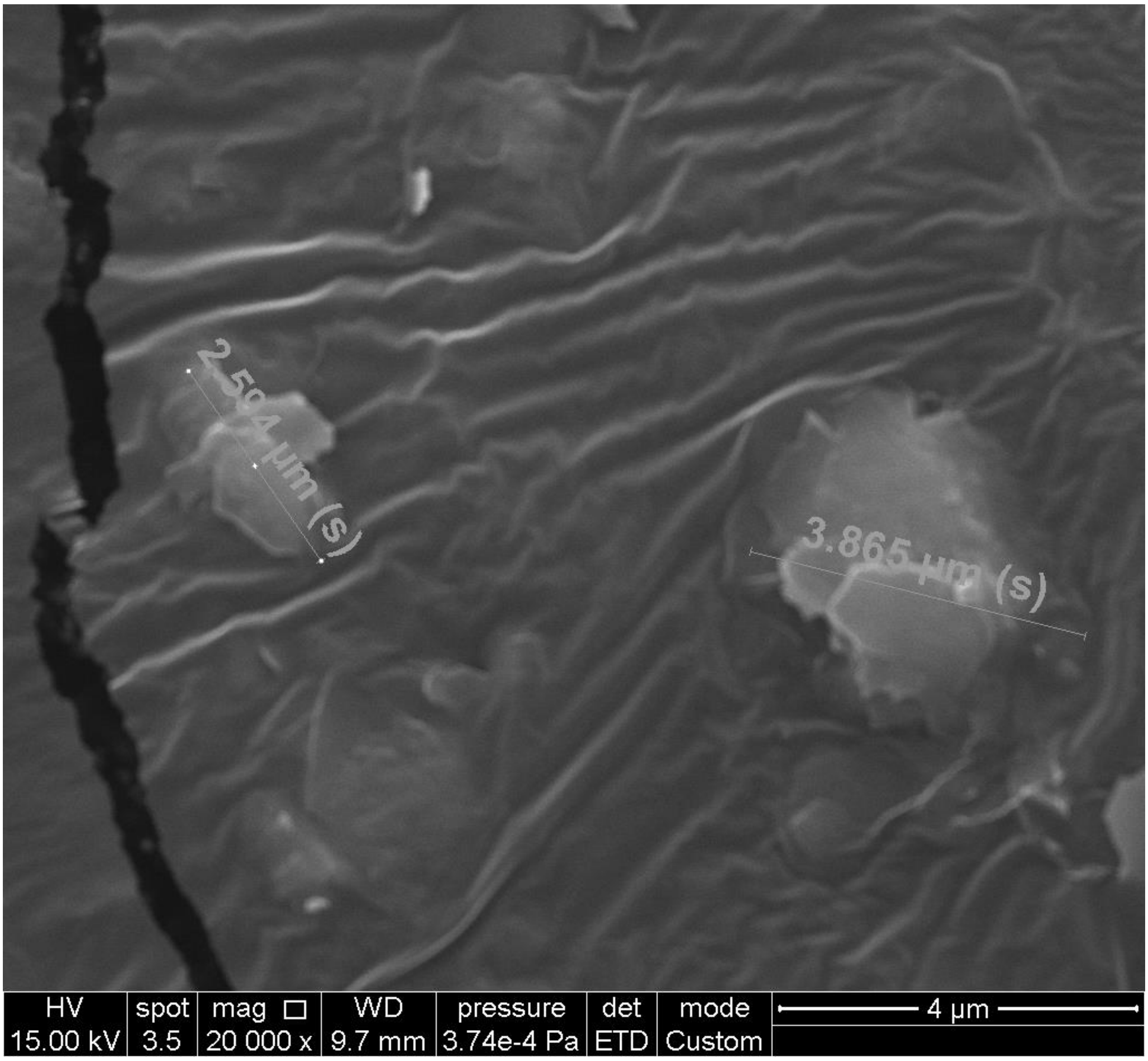
2.594 µm (s)
3.865 µm (s)
HV
15.00 kV
spot
3.5
mag
20 000 x
WD
9.7 mm
pressure
3.74e-4 Pa
det
ETD
mode
Custom
4 µm

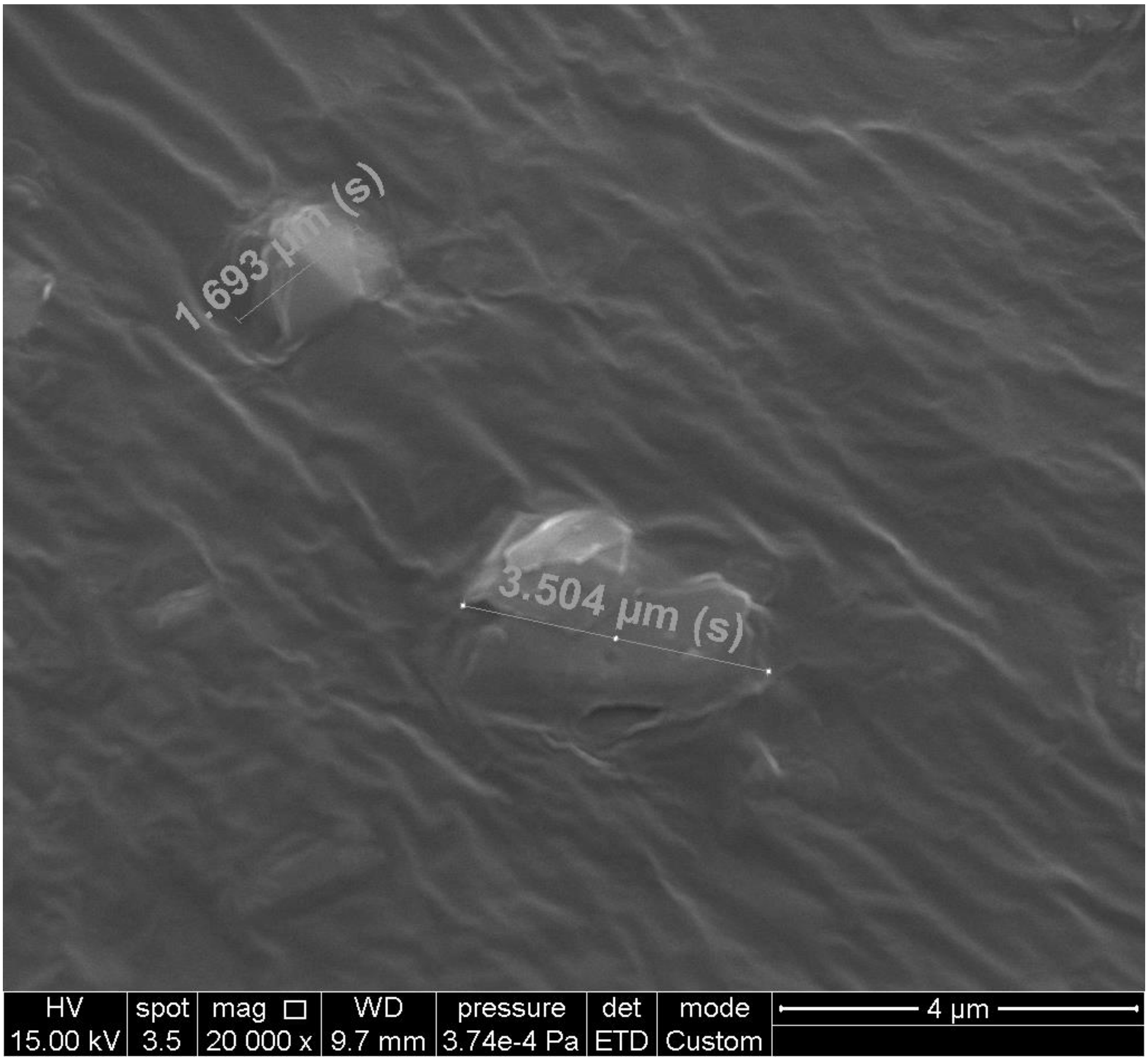
1.693 µm (s)
3.504 µm (s)
HV 15.00 kV
spot 3.5
mag 20 000 x
WD 9.7 mm
pressure 3.74e-4 Pa
det ETD
mode Custom
4 µm

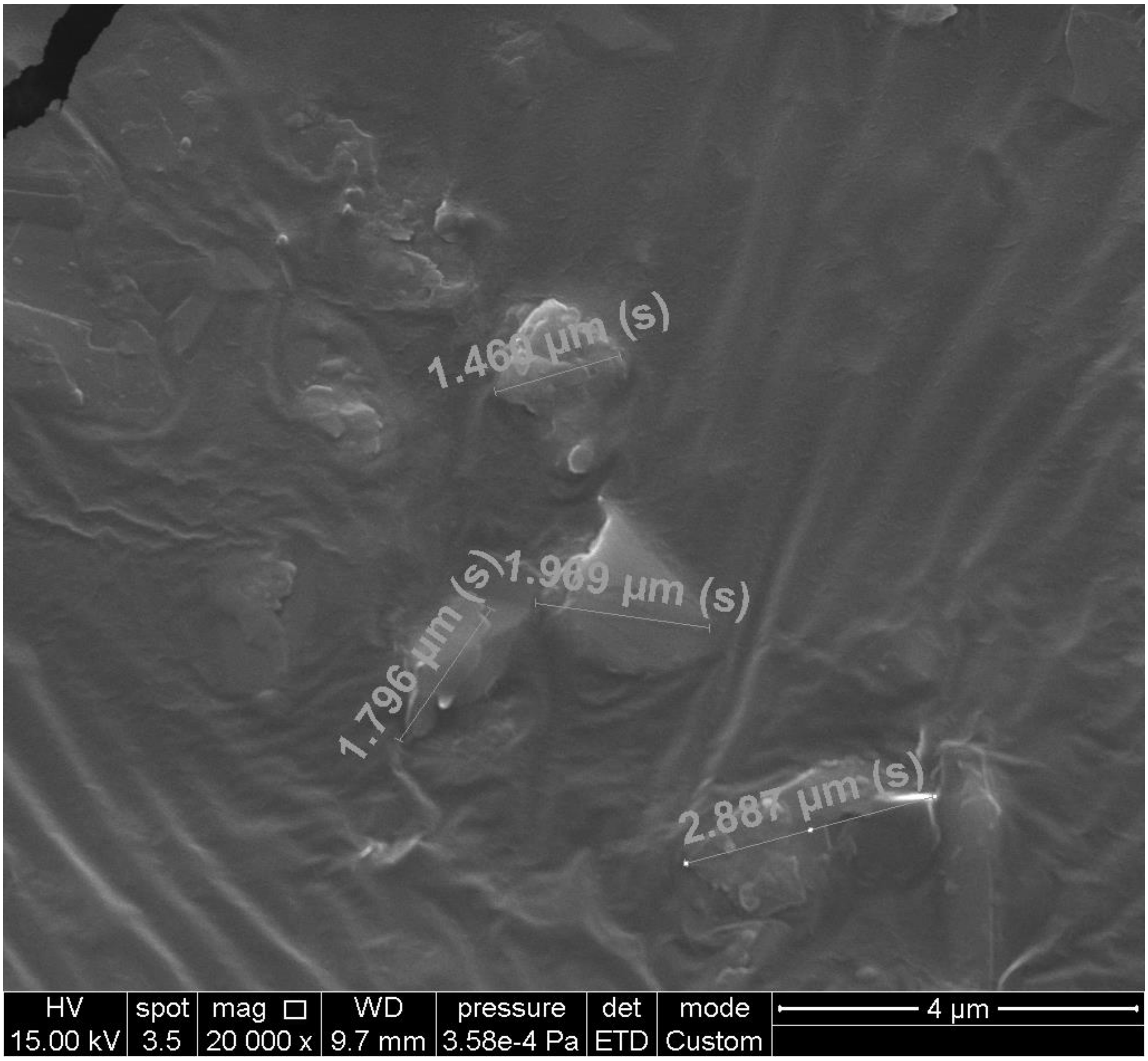
1.796 µm (s)
HV
15.00 kV
spot
3.5
mag
20 000 x
WD
9.7 mm
pressure
3.58e-4 Pa
det
ETD
mode
Custom
4 µm

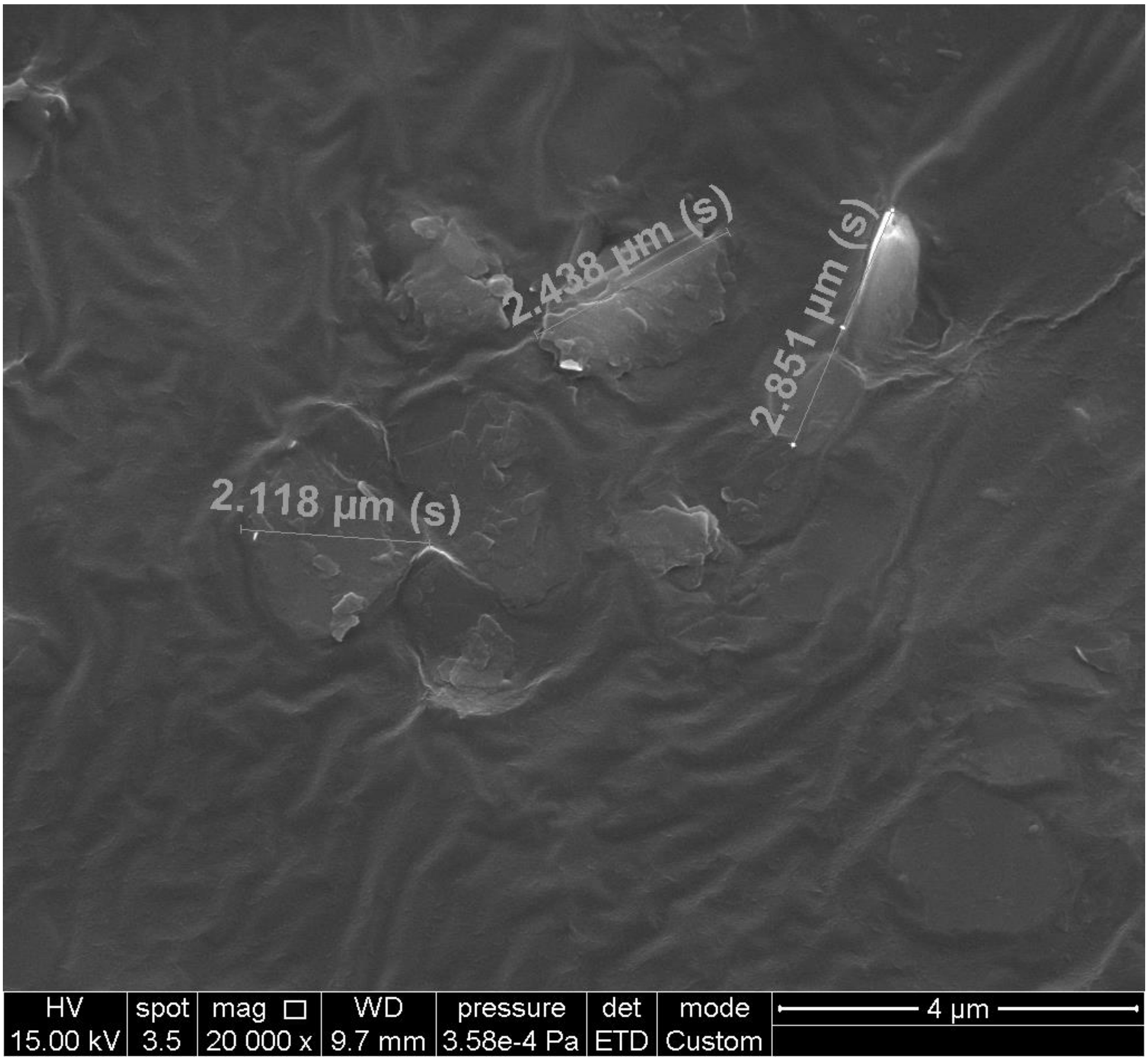

2.438 µm (s)
2.851 µm (s)
2.118 µm (s)
HV | spot | mag | WD | pressure | det | mode
15.00 kV | 3.5 | 20 000 x | 9.7 mm | 3.58e-4 Pa | ETD | Custom
4 µm

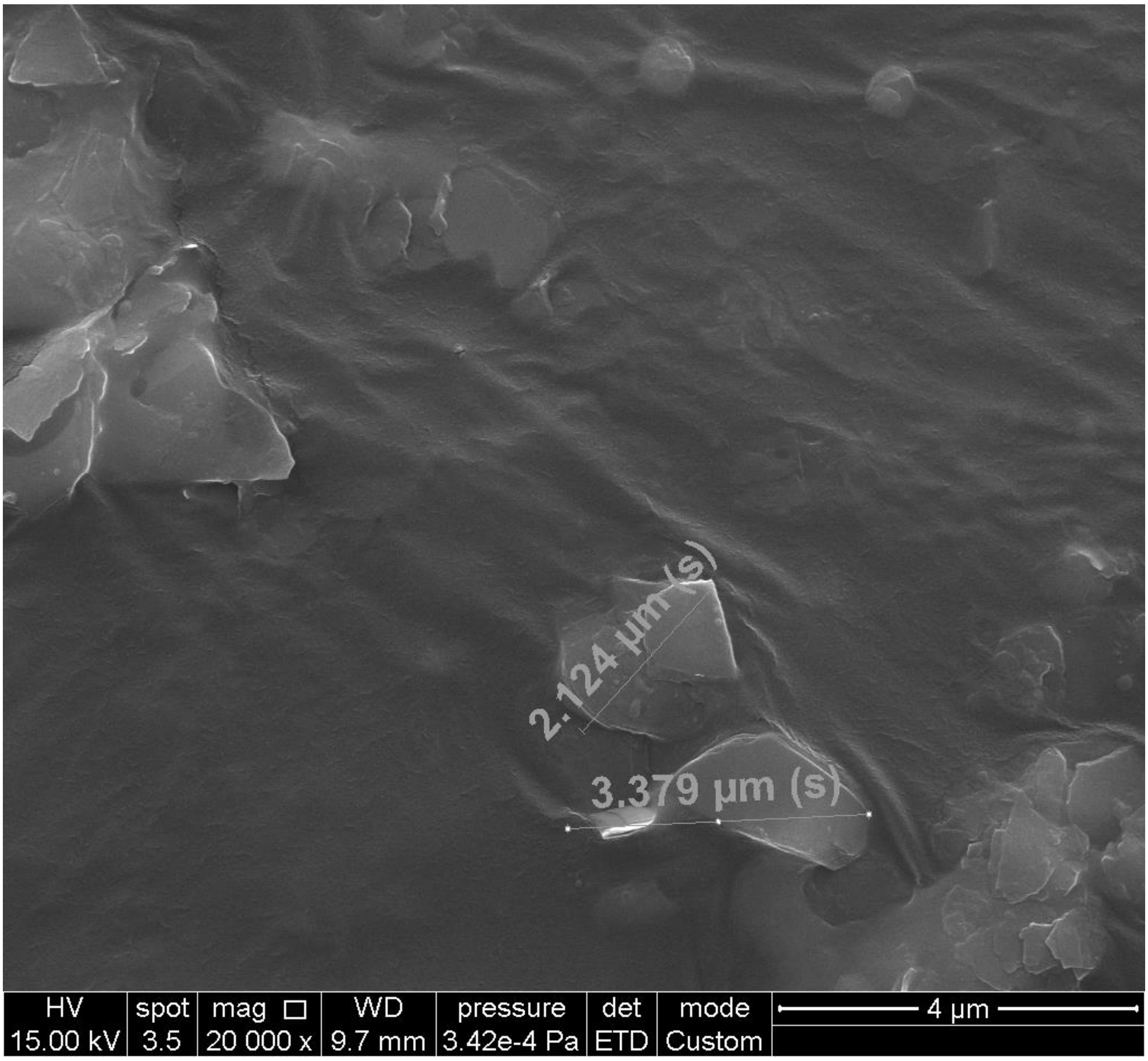
2.124 µm (s)
3.379 µm (s)
HV
15.00 kV
spot
3.5
mag
20 000 x
WD
9.7 mm
pressure
3.42e-4 Pa
det
ETD
mode
Custom
4 µm

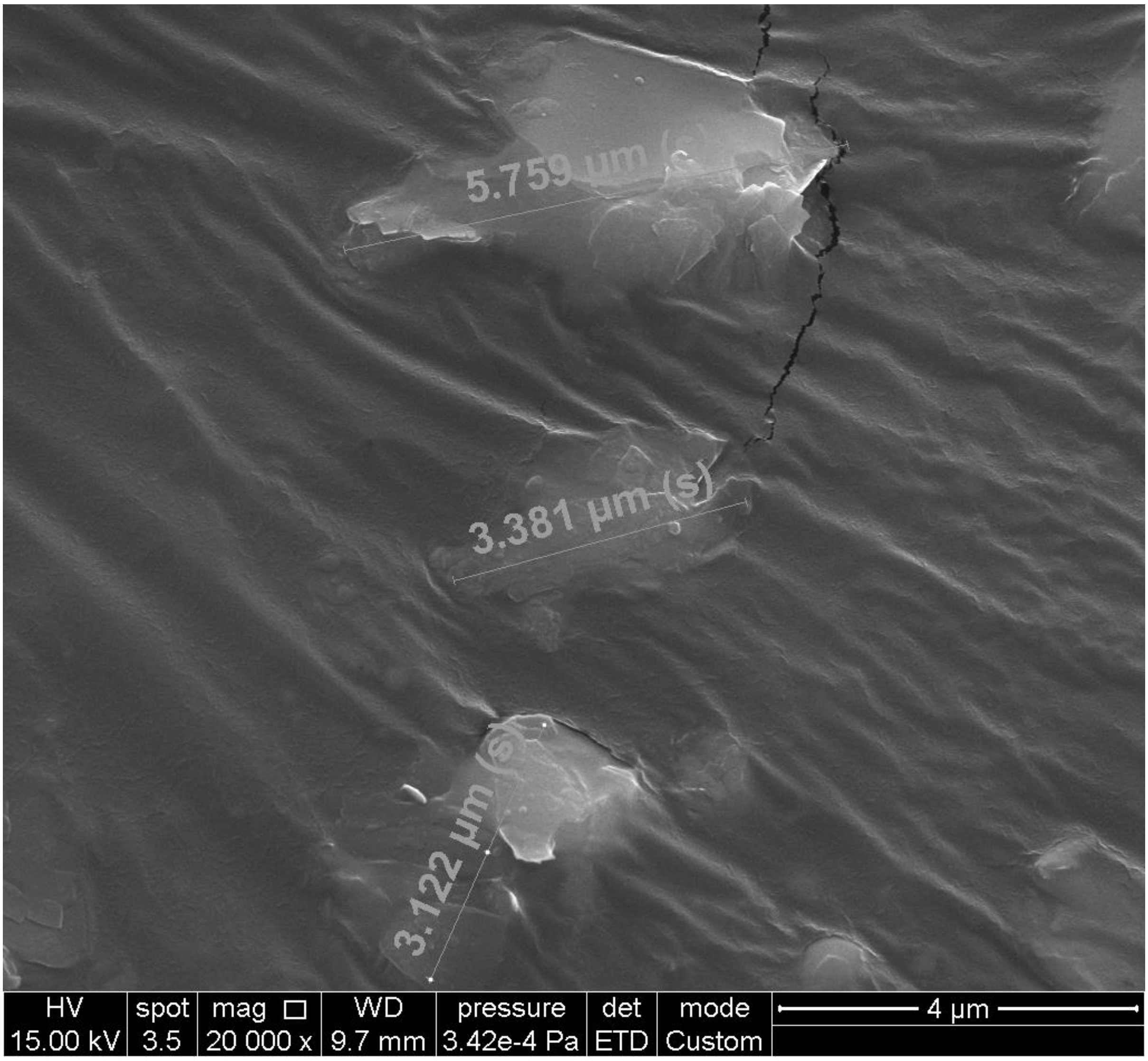
5.759 µm (s)
3.381 µm (s)
3.122 µm (s)
HV
15.00 kV
spot
3.5
mag
20 000 x
WD
9.7 mm
pressure
3.42e-4 Pa
det
ETD
mode
Custom
4 µm

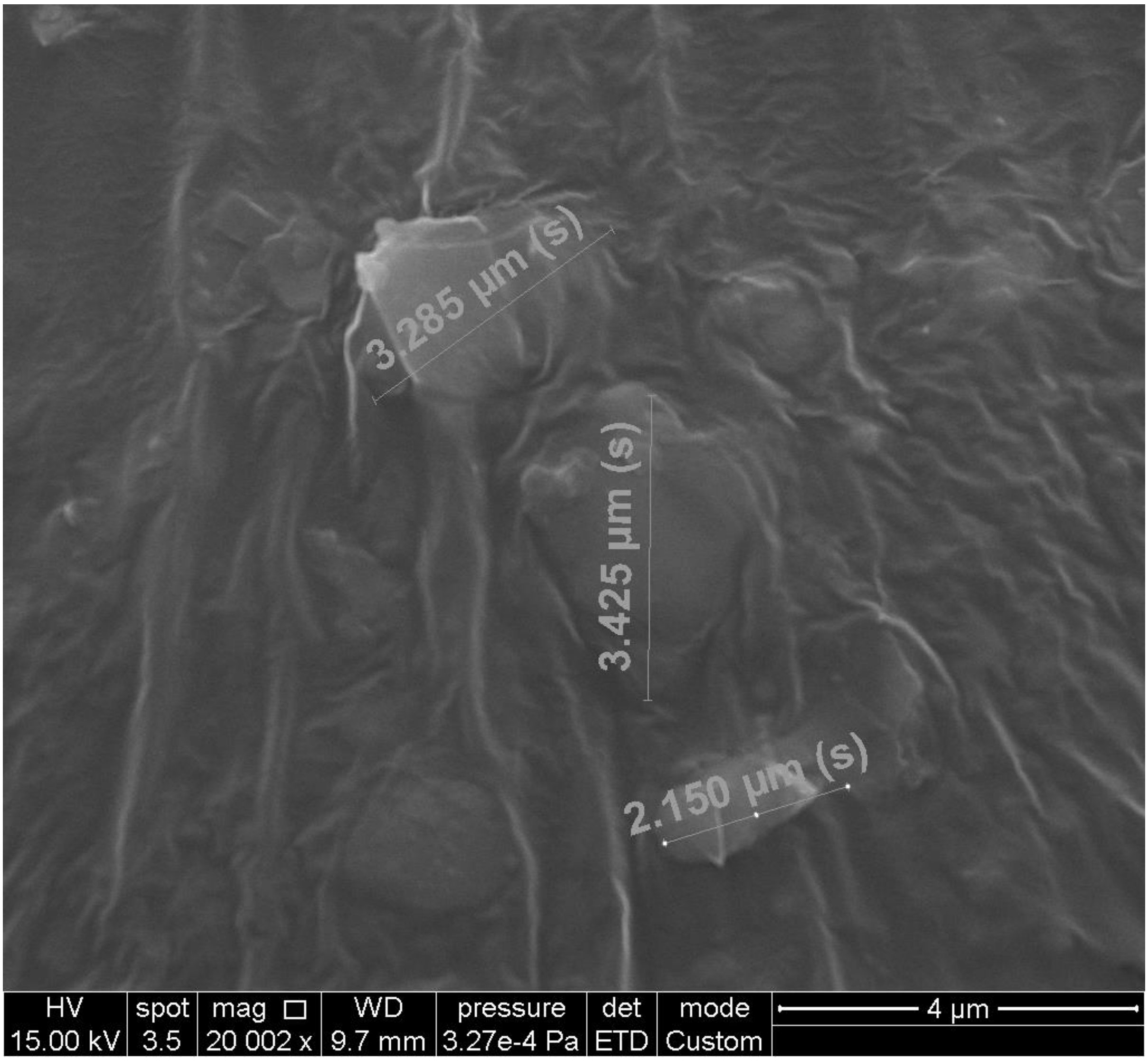
3.285 µm (s)
3.425 µm (s)
2.150 µm (s)
HV
15.00 kV
spot
3.5
mag
20 002 x
WD
9.7 mm
pressure
3.27e-4 Pa
det
ETD
mode
Custom
4 µm

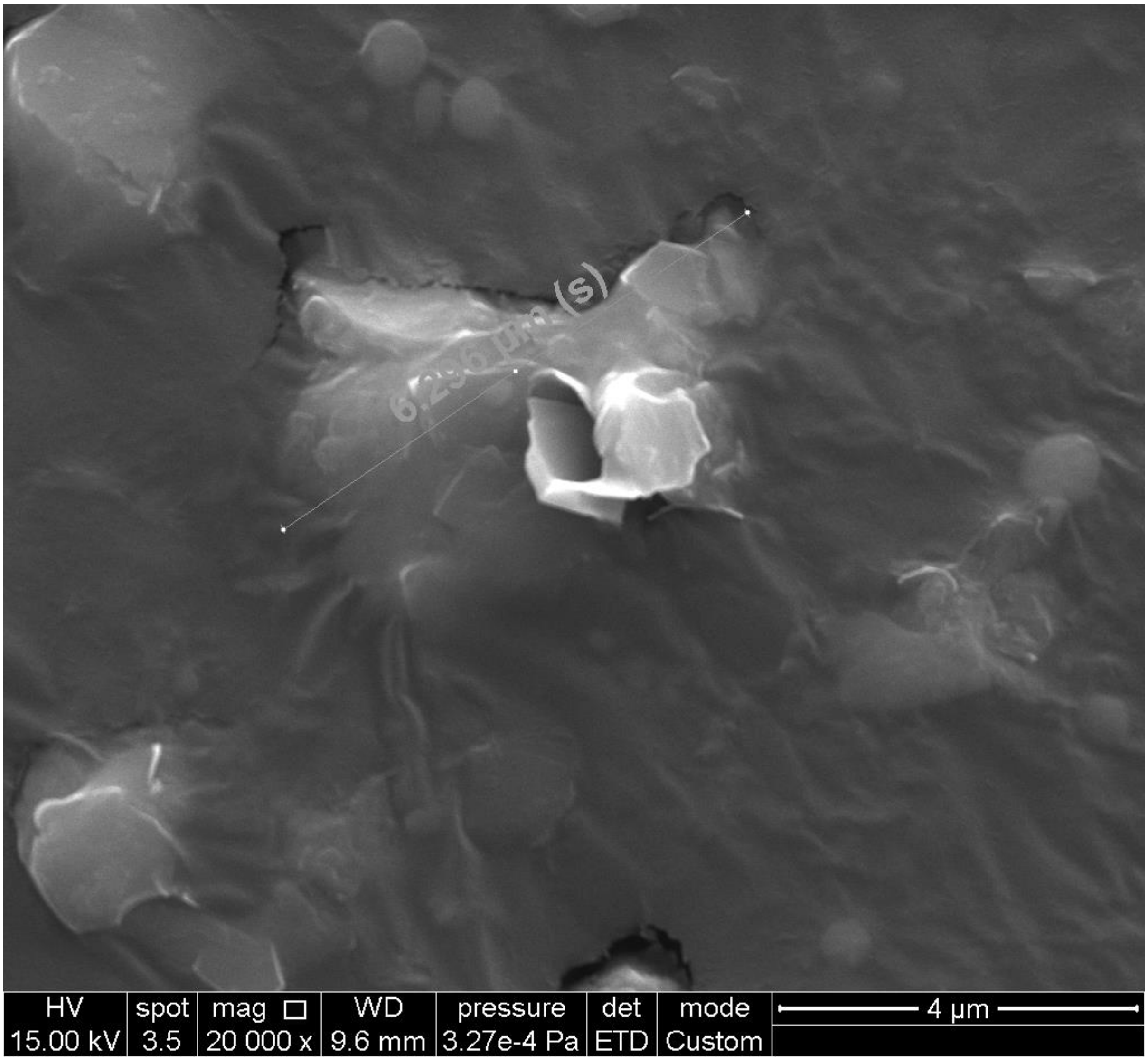
6.296 µm (s)
HV
15.00 kV
spot
3.5
mag
20 000 x
WD
9.6 mm
pressure
3.27e-4 Pa
det
ETD
mode
Custom
4 µm

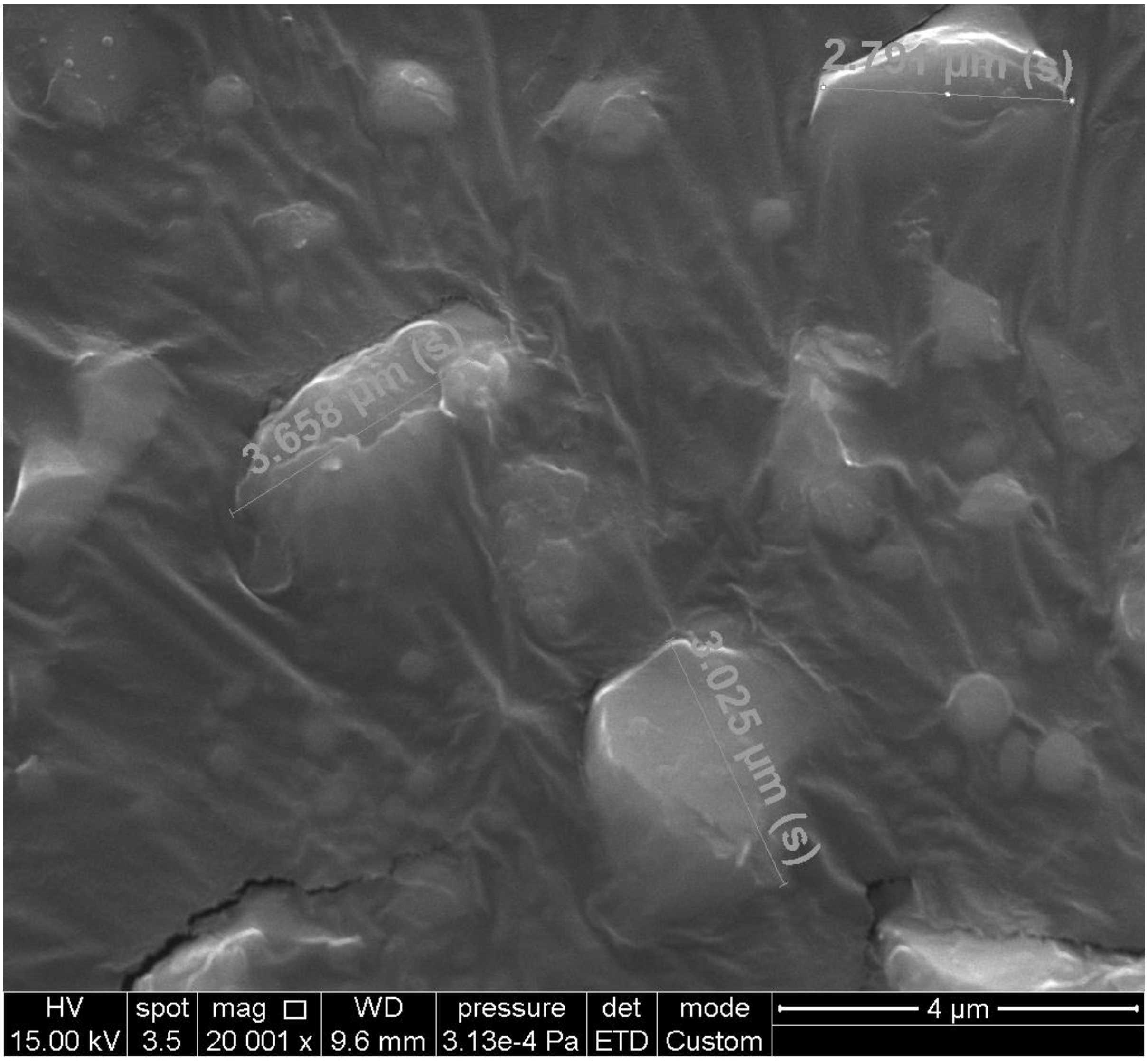
3.658 µm (s)
3.025 µm (s)
HV
15.00 kV
spot
3.5
mag
20 001 x
WD
9.6 mm
pressure
3.13e-4 Pa
det
ETD
mode
Custom
4 µm

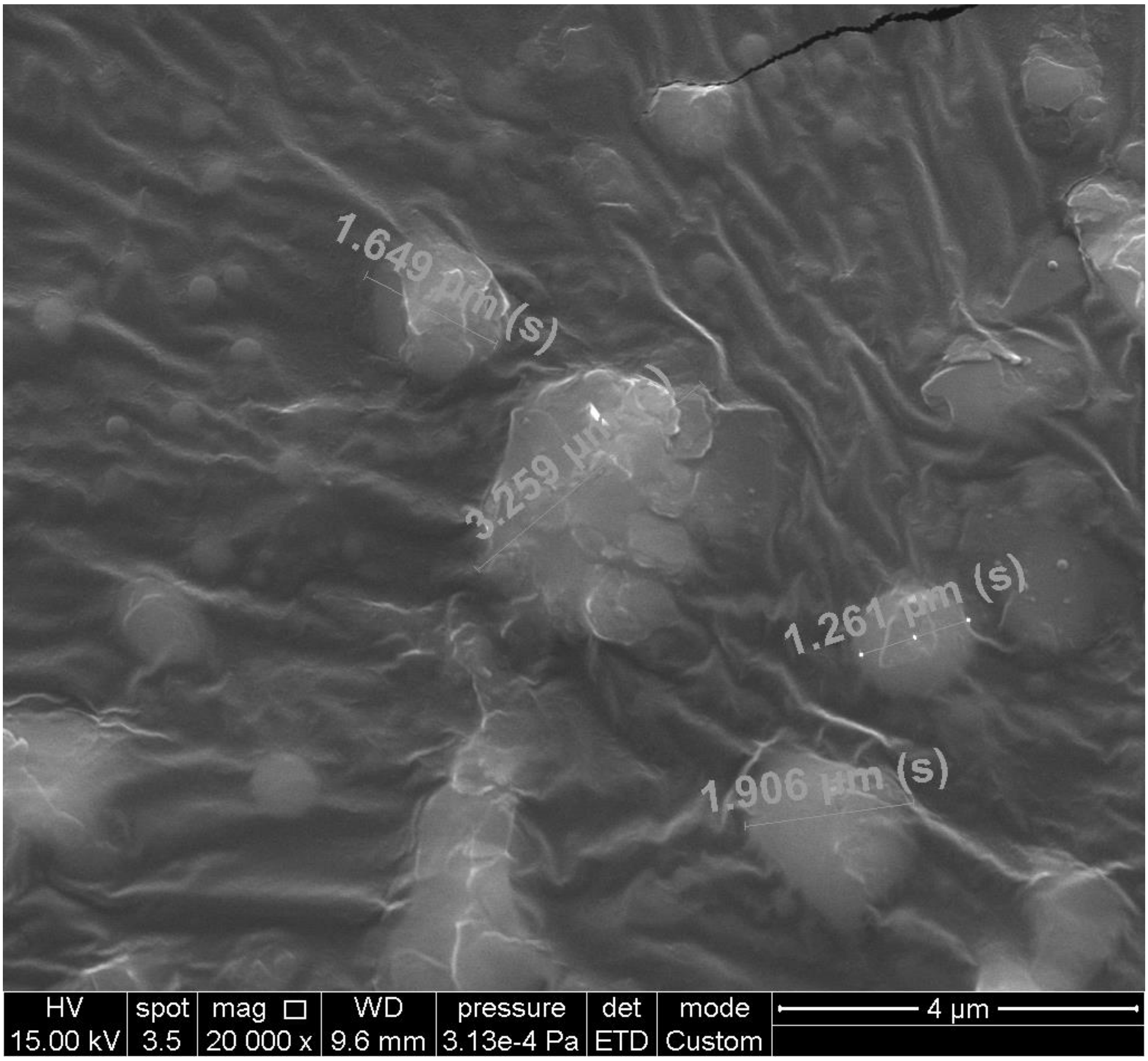
1.649 µm (s)
3.259 µm (s)
1.261 µm (s)
1.906 µm (s)
HV 15.00 kV
spot 3.5
mag 20 000 x
WD 9.6 mm
pressure 3.13e-4 Pa
det ETD
mode Custom
4 µm

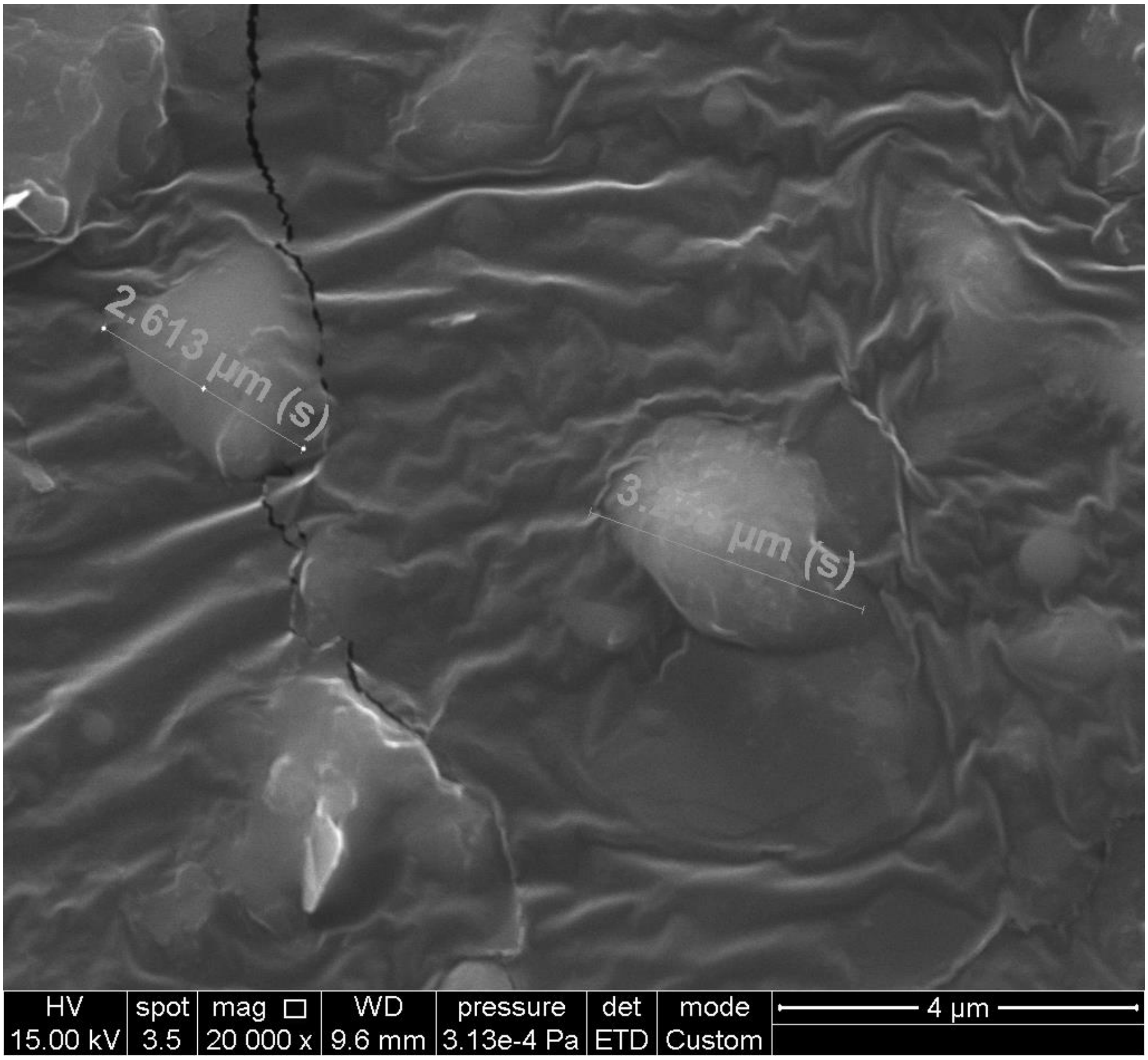
2.613 µm (s)
HV
15.00 kV
spot
3.5
mag
20 000 x
WD
9.6 mm
pressure
3.13e-4 Pa
det
ETD
mode
Custom
4 µm

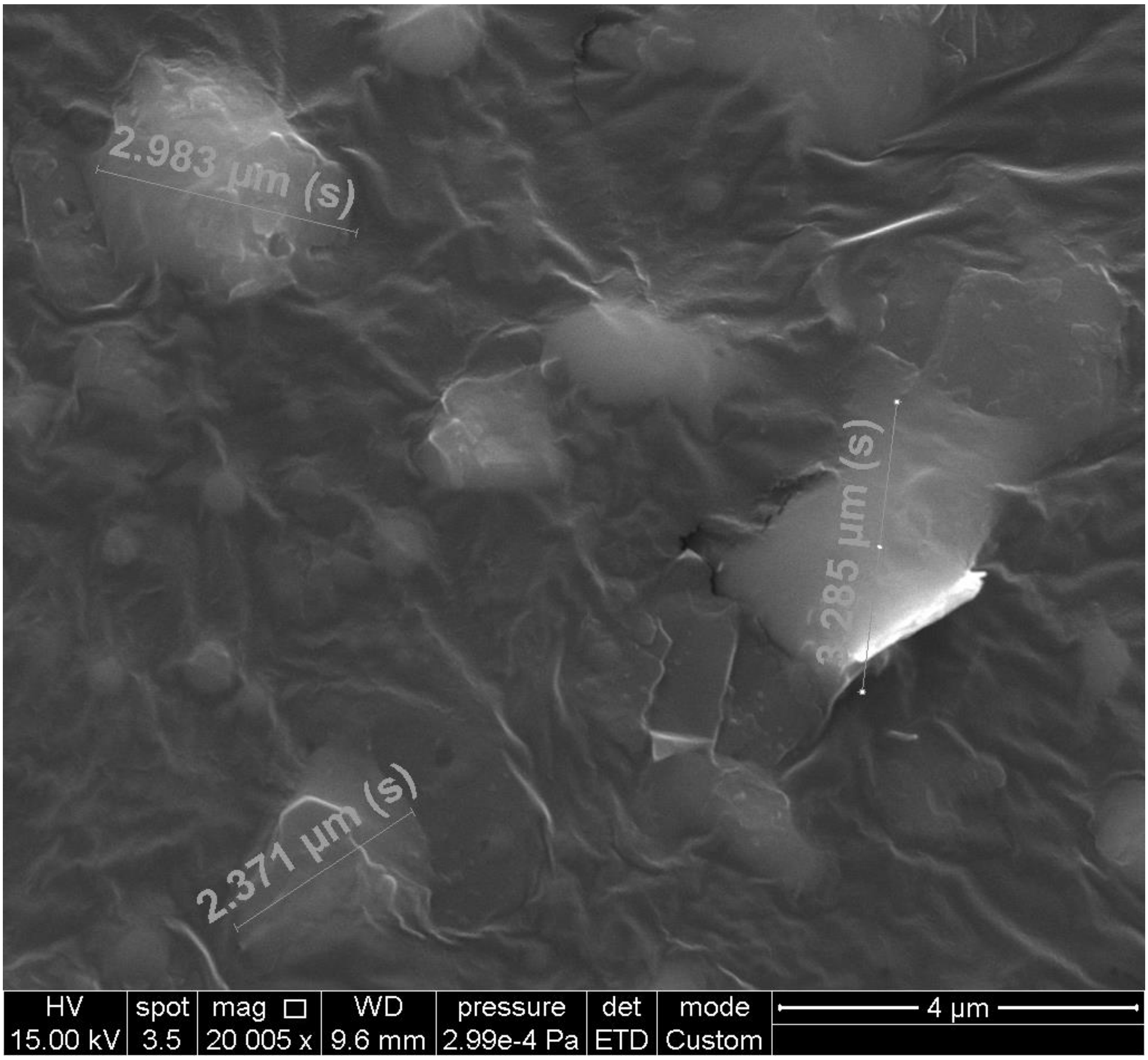
2.983 µm (s)
3.285 µm (s)
2.371 µm (s)
HV 15.00 kV
spot 3.5
mag 20 005 x
WD 9.6 mm
pressure 2.99e-4 Pa
det ETD
mode Custom
4 µm

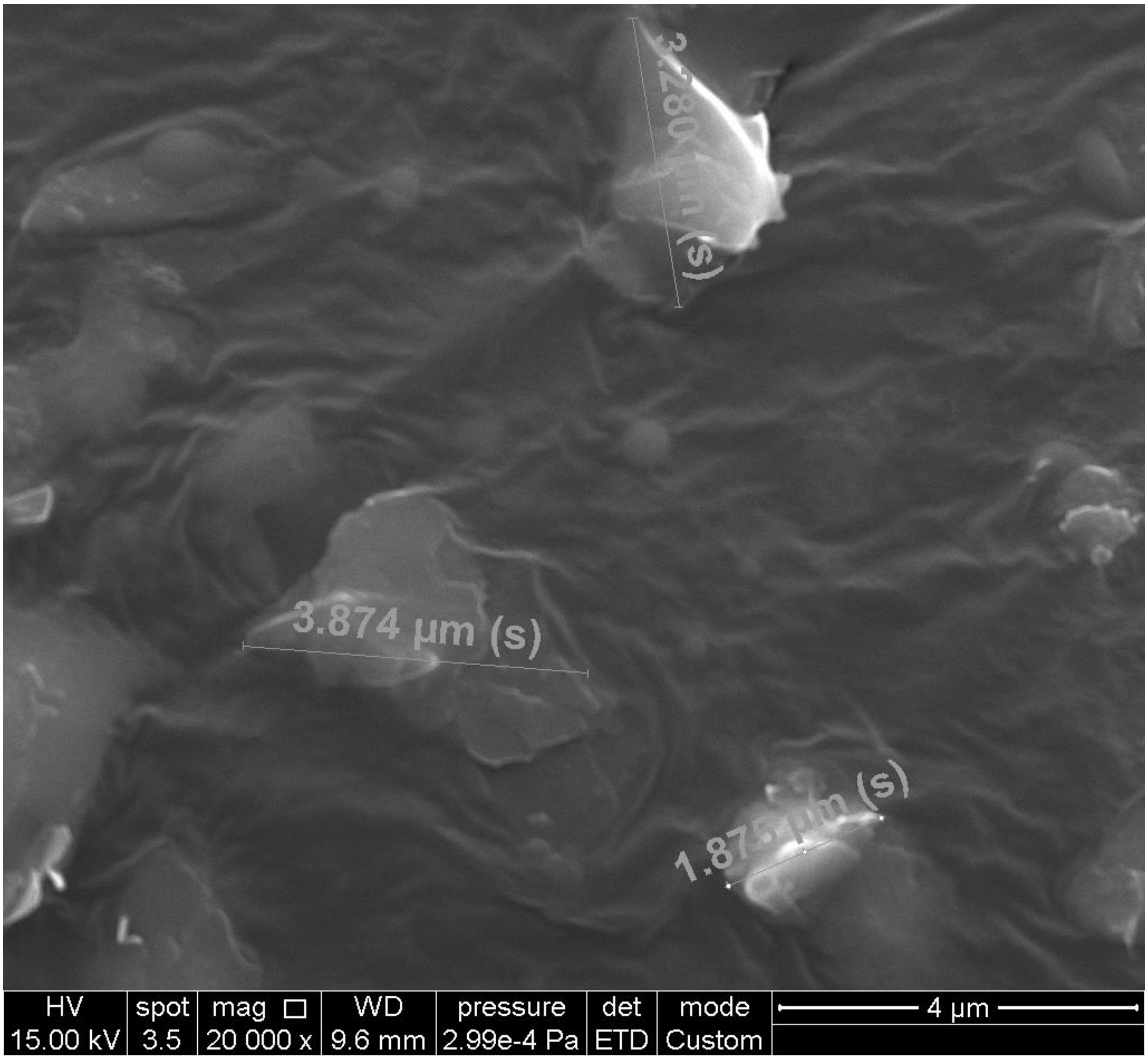
3.874 µm (s)
HV
15.00 kV
spot
3.5
mag
20 000 x
WD
9.6 mm
pressure
2.99e-4 Pa
det
ETD
mode
Custom
4 µm

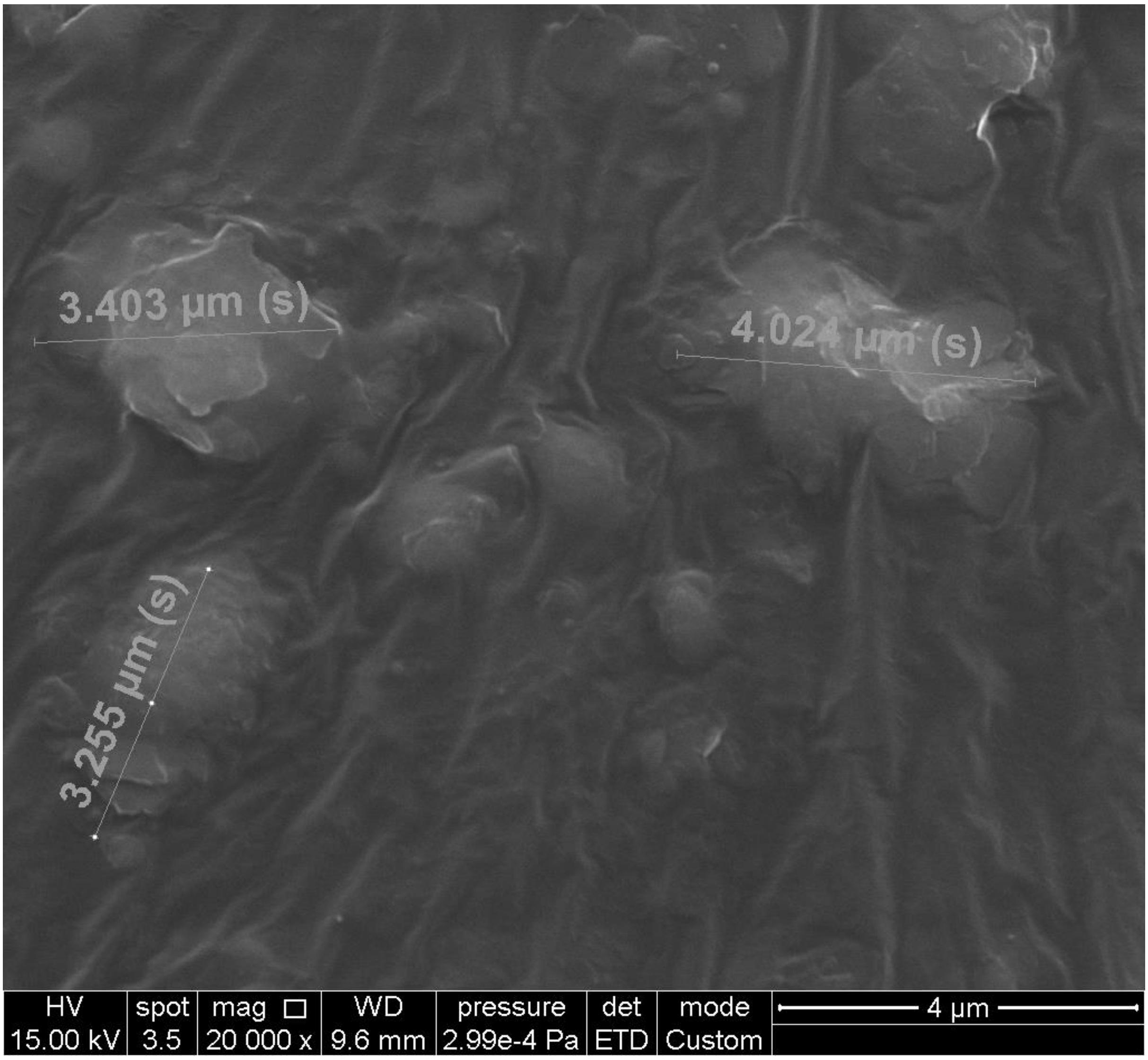
3.403 µm (s)
4.024 µm (s)
3.255 µm (s)
HV
15.00 kV
spot
3.5
mag
20 000 x
WD
9.6 mm
pressure
2.99e-4 Pa
det
ETD
mode
Custom
4 µm
